# Radio Telemetry and Harmonic Radar Tracking of the Spotted Lanternfly, *Lycorma delicatula* (White) (Hemiptera: Fulgoridae)

**DOI:** 10.3390/insects15010017

**Published:** 2023-12-30

**Authors:** Matthew S. Siderhurst, Kelly M. Murman, Kyle T. Kaye, Matthew S. Wallace, Miriam F. Cooperband

**Affiliations:** 1 Department of Chemistry, Eastern Mennonite University, Harrisonburg, VA 22802, USA; 2 Forest Pest Methods Laboratory, USDA-APHIS-PPQ-S&T, Buzzards Bay, MA 02542, USA; 3 Biology Department, East Stroudsburg University, East Stroudsburg, PA 18301, USA; mwallace@po-box.esu.edu

**Keywords:** movement, step distance, turning angle, host trees, aggregation, phenology, preferred height, invasive species

## Abstract

**Simple Summary:**

The spotted lanternfly is an invasive insect that damages a number of economically important plants and is a nuisance pest for residents and businesses. An improved understanding of spotted lanternfly movement may lead to better control methods for this pest that help slow its spread. To better understand spotted lanternfly movements, two types of tracking technologies were tested: radio telemetry and harmonic radar. A tag was attached to each spotted lanternfly which was then released into the wild and tracked. Both the adults and fourth-instar nymphs were tracked during the course of this study. More than half of the tracked adults remained sedentary or moved less than 5 m, whereas active adults moved up to 434 m, averaging under 20 m per 1–3 d period, and activity patterns varied by sex and adult stage. The longest distances were moved by females between the first field observation of mating and two weeks after the first fresh egg mass was found, and male movements continued increasing for an additional two weeks. SLF height in trees at the beginning of a movement was significantly and positively related to the distance of the subsequent movement. Most adults were found 6–9 m high in trees. During mating time, tracked SLF were significantly higher than 8 m and oriented to trees where tight aggregations of SLF already occurred. Tracked nymphs were found to walk almost 30 m over a five-day period.

**Abstract:**

*Lycorma delicatula* (White) (Hemiptera: Fulgoridae), spotted lanternfly (SLF), is an invasive pest that feeds and oviposits on numerous woody and herbaceous plants important to agricultural, forest, ornamental, and nursery industries. Describing and understanding SLF movements is key to implementing surveillance and control strategies for this pest and projecting population spread. We used radio telemetry (RT) and harmonic radar (HR) to track the movements of individual SLF at field sites in eastern Pennsylvania and northwestern New Jersey. SLF equipped with HR or RT tags were tracked in 2019 and 2020 from adult emergence until oviposition time, and their movements are described. Although the bulkier RT tags disproportionately affected the distance traveled by males, which are smaller than females, both males and females were more likely to be lost due to signal attenuation when affixed with the lighter-weight HR tags. Females were tracked moving longer distances than males, with maximum distances of 434 m by a single female and 57 m by a single male. A significant positive relationship was found between their height in trees and the distance of subsequent movement. Adult SLF were found in trees predominantly at heights between 6–9 m high. For the fraction of SLF found at eye level, males, but not females, significantly moved above eye level in the weeks prior to mating, likely resulting in the observed sex ratio shift that defines the Early-2 stage. During mating time, tracked SLF were significantly higher than 8 m and oriented to trees where tight aggregations of SLF were present. This orientation towards tight aggregations started when mating began and peaked in the following 2.5 weeks for males in Late-1 and the beginning of Late-2 (after oviposition began), whereas females started this orientation behavior a half-week after males, and this activity peaked for two weeks. Male and female SLF adults exhibited slight differences in host preference, and strong preferences for wild grape, black walnut, sweet birch, and tree-of-heaven were observed. The HR-tagged nymphs moved up to 27.6 m over a five-day period in a cornfield. Nitinol wire HR tags performed better than Wollaston process or tungsten wire tags. SLF movement parameters in the field are described.

## 1. Introduction

The spotted lanternfly (SLF) *Lycorma delicatula* (White) is an invasive pest insect present in at least 15 eastern US states [1,2]. Native to China [3], SLF was first discovered in Berks County, Pennsylvania in 2014 [4] and has since spread to several other states. Although their movements result in a natural population spread from their introduction point, SLF are most likely to spread via human-assisted movement [5]. A dead female *L. delicatula* was discovered in a shipment sent from Pennsylvania to an Oregon nursery [6], and models suggest SLF may reach and establish in West Coast states within a decade [7,8]. SLF has a wide host range of over 70 species including economically important species such as grapevines, apple trees, stone fruit trees, ornamental trees, and valuable hardwoods [3]. As a generalist sap feeder, SLF causes direct damage to affected plants through feeding, thereby reducing plant nutrients [9,10]. Indirect damage may also result from feeding wounds that facilitate pathogen transmission [9] and decreased photosynthetic ability [11,12] due to sooty mold growth that results from excreted SLF honeydew [13]. While SLF has produced severe adverse effects for the multibillion-dollar grape, tree fruit, timber, and ornamentals industries in the affected states [14], its impact as a nuisance pest is also notable. Large numbers of SLF can impact outdoor enjoyment and activities by their mere presence, and also by coating outdoor structures, recreational items, and furniture with honeydew and sooty mold which is unsightly and attracts other insects that feed on it, such as bees, wasps, and ants.

The life cycle and phenology of SLF in the area bordering eastern Pennsylvania and northern New Jersey have been previously described [2,15,16]. First-instar SLF nymphs typically emerge from their egg masses around the last week of May, at which time they begin to feed and develop, molting approximately every two to three weeks through four nymphal instars. Stages are relatively well-synchronized. Around the end of July, fourth-instar SLF start to molt into adults. Adult SLF are relatively long-lived, with the time between emergence and death being approximately 10–16 weeks. In earlier work, we broke down their long adult stage into three phases based on their physiological state, changing ecology, and behavioral activities [17], and more recently we have further refined these phases and their descriptions into two- to three-week periods based on our improved understanding of their phenology and physiological states [15]. Early-1 encompasses the first 2–3 weeks post-eclosion when adult SLF are focused on feeding and their sex ratio is approximately 50:50 on trunks of mature tree-of-heaven *Ailanthus altissima* (Mill.) Swingle (Sapindales: Simaroubaceae), their preferred adult host also native to China. Early-2 is marked by an observed sudden shift in sex ratio and encompasses a period of roughly 2–3 weeks in which they are observed in large, mostly female aggregations feeding heavily on *A. altissima*. Although during Early-2 the larger *A. altissima* trees can be found with mostly females on them, the sex ratio on other trees at this time can be mostly male [17,18]. After about two weeks of males being scarce on larger *A. altissima* trees, they suddenly and inexplicably reappear and the sex ratio reverts back to roughly 50:50. Then the first observation of mating in the field marks the beginning of Mid, which usually occurs around the first or second week in September in eastern Pennsylvania. Mid represents the period of time between the first observation of mating and the first observation of oviposition, but the time when peak mating occurs has not been well documented. The first observation of newly deposited egg masses marks the start of Late-1, which usually commences about a week after Mid, and also likely includes continued mating activities [15]. As with mating, the precise timing of when peak oviposition occurs has not been well documented. Late-2 begins two weeks after Late-1, during which time mating continues but tapers off while oviposition is also taking place. During Late-1, the sex ratio on *A. altissima* has been observed to gradually shift to a male bias which is sustained during Late-2 [15]. Two weeks after Late-2 is when Late-3 begins in which the sex ratio reverts back to 50:50, and oviposition is thought to be the primary activity before death, which usually occurs around the time of the first hard freeze, around early November.

SLF control presents numerous challenges because SLF populations move around between forested, residential, and agricultural areas [2]. Thus, control efforts aimed at reducing a population in an agricultural field may be defeated when new populations move in from other areas. Control regimes using insecticides [19,20] or *Beauveria bassiana* (Bals.-Criv.) Vuill. (Hypocreales: Cordycipitaceae) [21] have shown short-term effectiveness at reducing pest levels. However, these treatments are expensive, must be reapplied frequently, and must contend with constant re-infestation originating from wild hosts surrounding treated areas [19,20]. Studying the movements of SLF may provide insights into how to optimize IPM control strategies for this pest as has been conducted for *Halyomorpha halys* Stål (Hemiptera: Pentatomidae) [22] and *Drosophila suzukii* (Matsumura) (Diptera: Drosophilidae) [23]. Additionally, SLF movement data will allow better modeling of pest populations to understand potential pest distribution, quarantine deployment, optimizing trapping networks, and predicting pest outbreaks [24,25]. Dispersal capacity (specifically step distance and turning angles), spatial distribution, and density of pest insects are particularly important for improving agent-based modeling [26,27,28,29]. While some agent-based models are not spatially-explicit, many increasingly sophisticated models rely on movement parameters, such as turning angles and step distances, for the targeted pest species.

A number of tracking techniques have been used to study insect movement including radio frequency identification (RFID), harmonic radar (HR), and radio telemetry (RT) [30]. Each technology has different advantages and tradeoffs. RT was first used to monitor flying insects by Sprecher-Uebersax and Durrer [31] and Hedin and Ranius [32] who studied the behavior of large beetles, although the development of the tracking techniques for insects began with the work of Hayashi and Nakane [33,34] on dobsonfly larvae, *Protohermes grandis* (Thunberg) (Megaloptera: Corydalidae). In comparison to the other two tracking techniques mentioned above, RT employs battery-powered tags that can be used to uniquely identify individual insects tracked from a distance. This is accomplished by using a hand-held antenna and receiver that listens for and recognizes the unique signal broadcast by the tag, with a range of up to several hundred meters. However, the added weight of the battery in the RT tags limits their use to larger insects. RT has now been used to track a taxonomically diverse series of generally large insects [35] including the successfully monitored hemipteran triatomine bugs [36] and Asian giant hornet *Vespa mandarinia* Smith (Hymenoptera: Vespidae) [37].

HR was first used to study insect movement by Mascanzoni and Wallin [38] and subsequently, a relatively larger number of hemipteran species have been tracked using this technique [30]. Examples of HR-tracked hemipterans include *Nezara viridula* (L.) (Pentatomidae) [39], *H. halys* (Pentatomidae) [40], and *Riptortus pedestris* (F.) (Alydidae) [41]. Other hemipterans have been studied for potential tracking such as SLF [42,43], and *Ricania* sp. (Ricaniidae) [43]. Hemipterans are often medium-sized insects and can more easily carry relatively small HR tags, which often weigh several milligrams. There are two components of HR, (1) a radar transceiver unit, which both emits a directional microwave signal and ‘listens’ for a reflected signal at twice the broadcast frequency, and (2) a diode tag that receives the original microwave signal and reemits a frequency-doubled signal [38]. HR units can be stationary ground-based [44] or mobile, which includes handheld units [45,46]. In addition to being small, HR tags are less expensive and don’t require a battery, resulting in a relatively long shelf-life and field-life. However, they have several drawbacks in comparison to RT: the HR detection range with a hand-held radar unit is shorter (max 75 m) than with RT (max 500 m), HR tags aren’t uniquely identifiable from a distance because they rely on a reflected signal rather than generating a unique signal, and they are highly sensitive to both tag orientation relative to the radar unit and influences from the terrain and vegetation. Previous studies have tracked insects, including several Hemipteran species [39], using handheld HR units manufactured for avalanche rescue by the RECCO corporation [38,47,48].

Herein we report on the field tracking of SLF using both RT and HR technologies for adults and HR for nymphs. We also evaluate and compare HR tags in which the reflective antenna carried by the insect was fabricated from different experimental materials.

## 2. Materials and Methods

### 2.1. Tracking Materials

Biotracker receivers and NanoPin tags (model SRX800-M2, 160–167.999 MHz, 3 s burst rate) and folding 3-element Yagi antennas (Lotek Wireless, Inc., Newmarket, ON, Canada) were used for RT tracking. Radio telemetry tags used for all RT experiments (Lotek Wireless, Inc., Newmarket, ON, Canada) transmitted at 166.420 MHz, had a 3 mm dia. × 12 mm length, and a 7 cm antenna. RT tags weighed 150 mg and each produced a unique signal that could be used to identify individual insects.

Harmonic radar transceivers (model R9, RECCO AB, Lidingö, Sweden) were used for HR tracking. Dipole harmonic radar tags were fabricated from a Schottky diode (RECCO AB, Lidingö, Sweden) and platinum (Wollaston process, 0.00254 mm diameter, Leico Industries, Inc., Lyndhurst, NJ, USA), nitinol (0.076 mm dia., Malin Co., Cleveland, OH, USA) or tungsten wire (0.071 mm dia., California Fine Wire Co., Grover Beach, CA, USA). Two 8 cm lengths of wire were attached to the diode with UV-activated adhesive (Bondic, Niagara Falls, NY, USA) so that each wire touched one of the diode contacts while avoiding the opposite diode contact and the other wire. Electrical connections between the wires and the diode contacts were secured using conductive silver paint (GC Electronics, Rockford, IL, USA). Fully assembled HR tags weighed approximately 15 mg. Because HR tags do not produce unique signals, each HR-tagged SLF also received a numbered marker (queen bee marking kit, BioQuip, Rancho Dominguez, CA, USA) for identifying individual insects once located.

### 2.2. Experiment 1: Radio Telemetry and Adults

**Figure 1:**
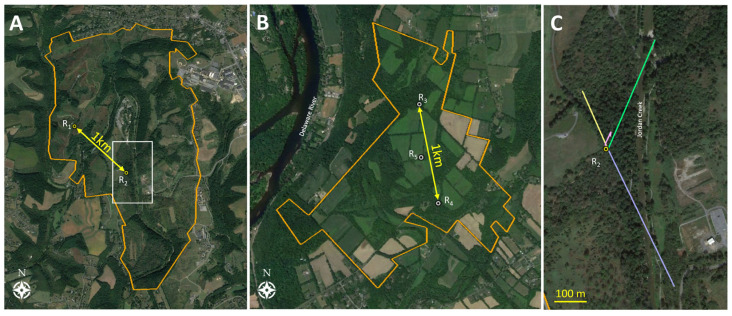
Satellite views of Trexler Nature Preserve used in 2019 (**A**) and Beaver Brook Wildlife Management Area used in 2020 (**B**), where Experiments 1 and 2 were conducted (parklands outlined in orange). Adult spotted lanternflies *Lycorma delicatula* (SLF) were tagged and released at designated release points (R). The two primary release trees at each site were approximately 1 km apart. The white box in Trexler Nature Preserve (**A**) is enlarged in (**C**) to depict the sample movements of four different SLF originating from release tree R_2_ (shown as blue, green, yellow, and pink travel vectors). The longest two vectors (shown in blue and green) represent two SLF that crossed over Jordan Creek, suggesting flight occurred with the RT tags (satellite images by Google Earth).

Experiment 1 investigated the movement of RT-tagged adult SLF released in natural settings dominated by mixed deciduous forest and edges, and interspersed with grassland/meadows or agricultural fields, and riparian ecosystems. The purpose of this experiment was to collect data on SLF movement directions, step lengths, height in the vegetation, and types of vegetation visited, and to observe if these parameters varied by sex or over time. Tracking work was conducted in 2019 and 2020 near the centers of two nature preserves located in Pennsylvania and New Jersey, respectively, each containing over 2 km^2^ of land (Figure 1). These areas were selected for being already infested with SLF, and for ease of tracking long distances in natural terrain away from fences or private properties.

In 2019, the study was located in Trexler Nature Preserve, Schnecksville, PA, USA (40.652402, −75.626655) which spans 4.45 km^2^. Two trees, spaced 1 km apart, were selected for releasing most of the RT-tagged SLF, and both release trees were the SLF preferred host plant, *A. altissima*. The area surrounding the first release tree, R_1_ (40.657, −75.634), which was on a hill, included open natural grasslands, forbs, shrubs, and occasional trees, with the nearest forest edge being at least 100 m away, so this release point is described as a “tree island” in a field habitat (Figure 1A). The second release tree at this nature preserve, R_2_ (40.651, −75.625), was a young tree located lower in elevation on the edge of a forested area to the southeast, adjacent to a natural field. The nearest known *A. altissima* to both release trees was another stand of *A. altissima* located 124 m and 928 m away from the northern (R_1_) and southern (R_2_) release trees, respectively. Additionally, a large stand of *A. altissima* with a high density of SLF on them was present 1.3 km north of the northernmost release point. In 2019, 50 males and 47 females affixed with RT tags (detailed below) were released on the two release trees. Mean prevailing wind direction and speed were obtained from a weather station located adjacent to the preserve (40.67, −75.59).

In 2020, 44 adult males and 44 adult females, affixed with RT tags, were released at three release sites located in the Beaver Brook Wildlife Management Area in Warren County, NJ, USA (40.855481, −75.036069), which is 2.78 km^2^. This area was composed of mature forest with a canopy approximately 6 m higher than the site in 2019. In the inner forest, which we defined as being out of the visual range of the forest edge, the first release site, R_3_ (40.863, −75.038), contained a shagbark hickory *Carya ovata* (Mill.) K. Koch (Fagales: Juglandaceae), where 8 males and 8 females were released during Early-1, and a sweet birch, *Betula lenta* L. (Fagales: Betulaceae) with a large (~3 cm DBH) wild grapevine, *Vitis* sp. (Vitales: Vitaceae), climbing epiphytically on it, where 10 females and 11 males were released during Early-2 and Mid. The second release point, R_4_ (40.855, −75.036), 1 km to the south and also in the inner forest, used a black walnut tree, *Juglans nigra* L. (Fagales: Juglandaceae), from which 16 females and 23 males were released during Early-1, Early-2, and Mid. The third release point halfway between the others, R_5_ (40.859, −75.038), used a black walnut tree on a forest edge to release 10 females and 2 males during Early-1 (Figure 1B). The vicinity surrounding the release sites included agricultural fields and forests, but unlike in 2019, *A. altissima* trees were present in the general vicinity, and there was a relatively low-density population of SLF on these trees at the beginning of the season. The mean prevailing wind direction and speed were obtained from a weather station located adjacent to the preserve (40.86, −74.99).

**Figure 2:**
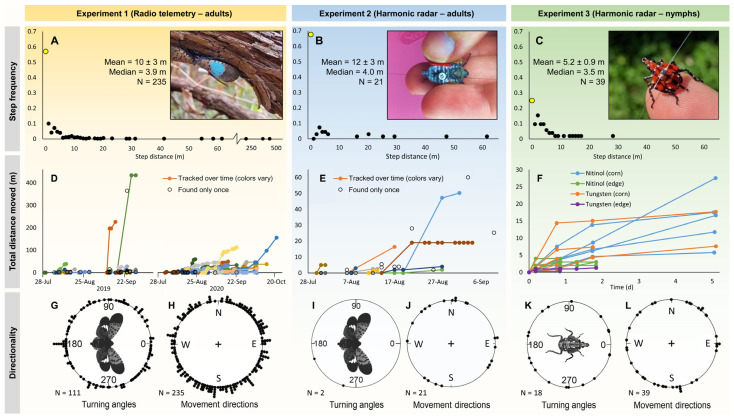
Spotted lanternfly, *Lycorma delicatula*, example tag attachments and step frequency for each experiment: Experiment 1 (**A**) (Photo Credit: Kelly Murman), Experiment 2 (**B**) (Photo Credit: Kyle Kaye), and Experiment 3 (**C**) (Photo Credit: Matthew Siderhurst). The HR-tagged adult (**B**) and nymph (**C**) are each shown with a nitinol wire tag. In (**A**–**C**), the points representing the frequency of step distances of zero m are highlighted with a yellow circle while all other distances are shown in black. The total distance moved for Experiments 1 (**D**), 2 ((**E**), data from both years combined), and 3 (**F**) are shown in the second row of graphs. Differently colored circles in (**D**,**E**) represent different individual tracked SLF. Turning angle and flight direction are shown for Experiments 1 (**G**,**H**), 2, (**I**,**J**), and 3 (**K**,**L**), respectively, with N representing the number of insects used in each calculation.

SLF adults were collected by hand in the vicinity of the study areas and held briefly in cages (30 cm × 30 cm × 30 cm, Bug Dorm, Megaview Science Co., Ltd., Taichung City, Taiwan) until tags were attached and the insects were released. RT tags were attached to adult SLF dorsally on the abdomen in a longitudinal orientation with the antenna pointing backwards (posteriorly) like a tail (Figure 2A), using cyanoacrylate adhesive (Rhino Ultra-Tough Glue, Rocklin, CA, USA). A tag attachment was conducted by two people: one held the insect and spread the wings while a second person applied the adhesive and positioned the tag until the adhesive cured. Care was taken not to glue the wings or the head during tag attachment. Once tagged, the wings were marked with bright paint markers (Posca P-5M, Mitsubishi Pencil Co., Ltd., Tokyo, Japan) to make them more visible from a distance (Figure 2A). This entire tagging and marking process took a few minutes after which they were flight-tested and released immediately.

Because of the size, shape, and weight of RT tags, each year, a portion of RT-tagged insects were flight-tested prior to their release to determine if they were flight-capable (gliding and directional changes). The initial flight tests helped us develop and adopt optimal methods of tag attachment, but even after protocols were established, we continued flight testing a subset of tagged insects. The testing protocol involved placing a tagged SLF on the end of a 12 m telescopic extendable pole. The pole was subsequently raised to a vertical position with the tagged SLF at its apex. The pole was then jiggled to induce the tagged insect to fall. The distance away from the pole that tagged insects traveled was recorded. Preliminary tests of various tag positions included the RT tags attached to the pronotum, the ventral abdomen, or with the antenna pointing forward (anteriorly), but those attachment positions were not adopted as they were observed to impede flight behaviors or other movements like walking in foliage. Pre-release flight testing was conducted on 11% and 81% of RT-tagged SLF prior to their release in 2019 and 2020, respectively.

During each release, a tagged SLF was placed onto the designated release tree trunk. Once released, tagged SLF were tracked five days per week until the detached tag or the dead tagged insect was located. Tracking and locating tagged SLF was accomplished by searching an area starting from the last recorded location and moving outward in a regular pattern. During searching, the Yagi antenna was moved slowly from side to side to maximize signal detection by aligning the receiver with the RT tag antenna on the SLF adult. Under optimal conditions, alignment of the RT tag antenna and receiver, without vegetation interference, had a maximum theoretical detection range of approximately 500 m. Due to physical and geographical features which would reduce this range, for determining search patterns a 100 m detection range was assumed. Binoculars were used to obtain visual confirmation when tagged subjects were located high in trees. Releases of RT-tagged SLF began in the last week of July and continued until the third week in September. Tracking activities continued through the end of the third week in October and included Early-1, Early-2, Mid, Late-1, and Late-2 phases.

### 2.3. Experiment 2: Harmonic Radar and Adults

Experiment 2 investigated the movement of HR-tagged adult SLF in the same field locations used in Experiment 1. SLF tracked with HR tags consisted of 23 females and 20 males in 2019, and 11 females and 6 males in 2020. The purpose of this experiment was to collect data on SLF movement directions, step lengths, height in the vegetation, and types of vegetation visited, and to observe if these parameters varied by sex or over time. In addition, we were also interested in comparing the two tracking techniques (RT and HR) in terms of data quality, longevity of tracking in the field, ease of use, encumbrance of SLF movement, and cost. In 2020, additional release trees were used, located far enough apart from each other that the HR-tagged SLF would be trackable separately. HR tags were attached to adult SLF on the dorsal abdomen in either a transverse (2019) or longitudinal (2019 and 2020) orientation (Figure 2B). Tag attachments were accomplished as in Experiment 1, with one person holding the insect while a second person applied the adhesive and positioned the tag until the adhesive cured. HR tags used during 2019 were fabricated with platinum wire, whereas in 2020, tags made with nitinol wire were used instead.

As with RT tags, pre-release flight testing was conducted on a subset (27%) of HR-tagged SLF to ensure tag attachment did not impede flight. Once released, HR-tagged SLF were tracked five days per week. Locating HR-tagged SLF was accomplished as described in Experiment 1 with RT-tagged SLF, with the following modifications. During searching, the RECCO unit was slowly rotated in the hand and moved from side to side to maximize signal detection by aligning the transceiver with the tag attached to the SLF. Under optimal conditions, alignment of the RECCO unit with the tag, without vegetation interference, had a maximum detection range of approximately 75 m. However, under field conditions with vegetation and suboptimal tag-transceiver alignment, the detection distance was appreciably attenuated. For determining search patterns, a 10 m detection range was assumed. Assuming this shorter detection range meant that HR-tagged SLF in tall trees, or those that moved long distances, were less likely to be located. Releases of HR-tagged SLF began in the last week of July and continued until 25 September. Tracking activities continued through 30 September.

### 2.4. Experiment 3: Harmonic Radar and Nymphs

Experiment 3 investigated the movement of HR-tagged fourth-instar SLF nymphs at a single location in PA. The purpose of this experiment was to collect data on SLF movement directions, step lengths, height in the vegetation, and types of vegetation visited, and to observe if these parameters varied between SLF tracked within a corn field vs. at the forest edge adjacent to the field. Additionally, the effect of wire type used to fabricate the HR tags on SLF movement was investigated. The nymph study site was located on a farm in southern Lancaster County, PA, USA (39.899489, −76.297267). SLF nymphs were collected by hand from a backyard garden on the outskirts of Ephrata, PA, USA, and transported to the study site in 30 cm^3^ collapsible screen cages (BioQuip, Rancho Dominguez, CA, USA). Nymphs had HR tags attached to the pronotum in a transverse orientation (Figure 2C) using a UV-activated adhesive (Bondic, Niagara Falls, NY, USA). Nymph tag attachments were conducted by a single person. Two types of release points were used in this study: (1) locations within a cornfield and (2) locations along a forest edge adjoining the cornfield. Release points were separated by a minimum of 20 m. Only one release (28 July 2020) was conducted with tagged SLF nymphs and tracking was undertaken for a week following the release. Search methods were identical to those described in Experiment 2.

### 2.5. Data Collection and Tracking Parameters

Each individual SLF that was tagged and released received a unique identification (ID) name and data sheet which was used to track and record its movement parameters every weekday. Upon release, recorded parameters included its ID, release date, technology (HR or RT), method of tag attachment, markings it was given to see it more easily from a distance, sex, release location, release tree species, whether or not it was flight tested prior to release, and if so, the distance it traveled from the flight test pole. Each time a tagged SLF was located, the parameters recorded on its data sheet included ID, date, status when found (alive/dead/tag detached), location (GPS coordinates), the plant species on which the tagged SLF was found, the density of naturally occurring SLF present on the same surface on which the tagged SLF was found (as estimated in the most heavily populated 10 × 10 cm^2^ area of trunk at eye level), the habitat type it was found (inner forest, forest edge, field), its height above ground in the vegetation as calculated either by triangulation using a laser range finder with slope measurement (Forestry Pro, Nikon, Zhejiang, China) or by averaging the visual estimates made by two different people using a reference of known length and counting how many lengths high the signal was, the distance and bearing from its last known coordinates (laser measurements of distance and bearing for short distances, or GPS for longer distances), a hand-drawn map of its incremental movement, and a section for additional note taking. Flagging was placed at the location where it was found to indicate where to start the next day’s search.

After the studies concluded, a survey of all woody plants within a 15 m radius of the three main release points (R_2_, R_3_, and R_4_) was conducted in the winter, and the frequency of each species (or genus, if more than one species was represented in a genus) was calculated. Although R_1_ was also a main release point, it had no woody vegetation to survey within 15 m. The frequency of SLF visits to each plant in relation to plant presence (plant-weighted visits) was calculated as

(1) $$\frac{v}{v+p}$$

where *v* is the frequency of visits, and *p* is the frequency of the woody plant in a 15 m radius around the release tree. To determine whether the frequency of plant visits differed by sex, the frequency of visits for each sex was used to scale the total number of steps to be equal for each sex while also keeping the total steps between them the same, then the scaled number of steps for each sex were compared using a chi-square test. Finally, when tagged SLF were able to be observed visually, their behaviors such as walking, resting, or courting were noted.

### 2.6. Statistics

Equations and regressions for linear relationships were calculated using Microsoft Excel, and linear regression analysis was conducted using JMP (v. 10.0.0). Turning angles and movement directions were analyzed using the Rayleigh test and the Hermans–Rasson test [49] to determine if directions of movement were random for each data set. Additionally, the V-test was used to assess the effect of wind direction on RT-tagged SLF movement directions in 2019 and 2020 [50]. Circular statistical analyses were performed using R packages CircStats, circular, and CircMLE [51]. Comparisons of SLF distances moved over time periods were conducted by analysis of variance (ANOVA) on ranked total distances moved per SLF, and log-transformed distances moved per step (a step is defined as an increment of time between two consecutive observations of an individual SLF), which normalized distribution and variance. The step-adjusted frequency of movement by females and males, for only steps that lasted 1–3 d, were analyzed using a contingency table approach with Pearson’s chi-square test to determine if differences in movement frequency occurred between stages (JMP v. 10.0.0). The step-adjusted rate of movement was calculated by dividing distance/time per step for non-zero-distance steps with tracking periods that lasted 1–3 d or 4–20 d. Differences in the step-adjusted rate of movement by stage, sex, and their interaction, were tested using standard least squares ANOVA and Tukey honestly significant difference (HSD) test for means separations on log-transformed rates (m/d), which normalized distribution and variance (JMP v. 10.0.0). Similarly, step-adjusted distances traveled in 1–3 d long steps were analyzed for males and females over their four physiological states using ANOVA and Tukey means separations on log-transformed distances, which normalized distribution and variance (JMP v. 10.0.0). The difference in heights at which SLF were found between the two years was analyzed using a Wilcoxon test. The total distribution of SLF steps among height groupings was tested using Pearson’s chi-square test, with the null hypothesis that the distribution was evenly distributed among all height groupings (JMP v. 10.0.0). For both height and density, the level that fell roughly midway among all observations was used as the cutoff point for chi-square comparisons over time. Chi-square tests were used to compare the frequency of steps occurring at heights above and below 8 m over time, and the frequency of steps occurring near higher and lower naturally occurring population densities over time, with expected frequencies being equal. An additional chi-square test was used to determine if the frequency of steps of males and females of each stage occurred at the seasonal expected frequency of 85% above eye level (2 m) and 15% below eye level, to test the hypothesis that the shifts in sex ratio at eye level during Early-2 reflect males and females moving upwards on trees at different times. Two-way chi-square tests were significant when the G-statistic was higher than 3.841 (α = 0.05) [52,53]. Comparisons of parameters between HR and RT, nitinol and tungsten, or corn and edge were made with *t*-tests unless data were not normally distributed, in which case the Wilcoxon test was used.

## 3. Results

### 3.1. Numbers of Insects, Steps, Stages, Courtship Observations, and Movements Tracked

In 2019 and 2020, the observed abrupt shift in sex ratio that marks the start of Early-2 occurred on 25 and 17 August, respectively. The first observation of mating, marking the start of Mid, occurred on 8 September in both years, and the first observation of fresh egg masses, the start of Late, occurred on 22 and 16 September in 2019 and 2020, respectively. Combining both years, tracking observations of adults with RT occurred between 20 July to 8 October (Early-1, Early-2, Mid, and Late), whereas for adults tracked with HR, observations were only between 31 July and 9 September (Early-1 and Early-2).

An overview of all the raw SLF movements (distance and frequency of steps, total distance moved per SLF, and directionality), from all three experiments can be seen in Figure 2. In total over the two years, 185 and 60 adult SLF were tagged and released with RT and HR tags, respectively, with 148 and 33 of those successfully tracked, and 116 and 20 of those having moved from their release position (Experiments 1 and 2, respectively). The total number of adult RT and HR tracking data points (steps) available for analysis in Experiments 1 and 2, respectively, were 535 and 62. Of those, 226 and 23 steps had movement (Table 1). There were 52 HR tracking data points generated in Experiment 3 on nymphs. For all SLF tracked in both years, trackers observed eight instances of tagged adult SLF engaged in courtship behavior, two observations occurred during Mid, four during Late-1, and two during Late-2.

### 3.2. Adult Step Duration and Frequency of Movement

**Figure 3:**
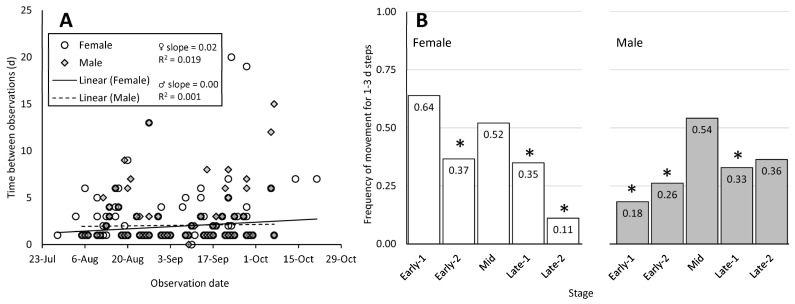
The period of time (days) between successive observations, or step duration, of individual adult spotted lanternflies (SLF), *Lycorma delicatula,* tracked with radio telemetry (Experiment 1), ranged from 1 to 20 d and varied over time, but SLF were located most often after a period of 1–3 d (**A**). Thus, standard-sized tracking periods of 1–3 d were used to calculate movement parameters. The frequency of movement (**B**) (when SLF moved from their previously known location as opposed to staying in the same place), over these 1–3 d tracking periods, changed significantly over five adult stages for both females (white) and males (gray) (asterisks indicate when observations with movement were significantly outnumbered by those without movement, Chi-square test, *p* < 0.05).

The time between two consecutive observations of the same adult SLF (step duration) ranged from 1 to 20 d for RT, and 1 to 11 d for HR, but most observations (89% and 72% for RT and HR, respectively) occurred 1–3 d after the previous observation (Figure 3A, Table 1). Importantly, outliers that were tracked over much longer periods of time had more opportunity to move both in potential number of movements made, and potential distance covered, but may not have moved during that time at all. Such outliers could potentially skew calculated parameters such as distance moved per step or rate of movement. Therefore, we controlled for differences in step duration by limiting analyses to only steps with standard 1–3 d durations. Thus, for the 533 RT steps used in movement calculations, we describe separately the analysis for 473 steps that lasted 1–3 d and 60 steps lasting 4–20 d. For distance calculations, these were further limited to the 226 steps that had movement, 183 of which were 1–3 d in duration, and 43 of which were 4–20 d in duration. By describing the steps that occurred over 1–3 d separately, we assess and describe SLF behaviors that took place during shorter time periods. Using 1–3 d steps, the proportion of SLF that moved, as opposed to remaining in place, changed significantly from expected even frequencies over time for both females (Pearson’s chi-square test, *p* < 0.001, χ^2^ = 18.98, N = 286, df = 4), and males (Pearson’s chi-square test, *p* = 0.034, χ^2^ = 10.42, N = 185, df = 4) (Figure 3B). Chi-square tests comparing the frequency of steps with and without movement at each stage found that Late-2 females (G = 12.40, N = 18), and Early-1 males (G = 4.82, N = 11) had significantly less movement, whereas Early-1 females (G = 2.81, N = 36), and Mid males and females had the highest frequencies of movement (females, G = 0.08, N = 48; males, G = 0.33, N = 48). Both sexes moved significantly less during the Early-2 (females, G = 7.31, N = 101; males, G = 9.92, N = 42) and Late-1 (females, G = 7.65, N = 83; males, G = 8.74, N = 73) stages flanking Mid (Figure 3B).

### 3.3. Distance and Rate of Adult Movement

All mean and individual step distances for adult SLF tracked with RT (Experiment 1) and HR (Experiment 2) are shown in Figure 2A,B. Step distances were categorized into 1 m intervals for that analysis. For all experiments, the most commonly recorded step distance was zero (i.e., tagged SLF did not move to a perceptibly different location during the current time interval) (Figure 2A–C). Total distances moved by individual adult SLF tracked with RT by observation date are shown in Figure 2D,E. Step distance might have been confounded by step duration in unfiltered raw data for the reason described in the preceding section.

#### 3.3.1. Distance of Adults with Radio Telemetry (Experiment 1)

**Figure 4:**
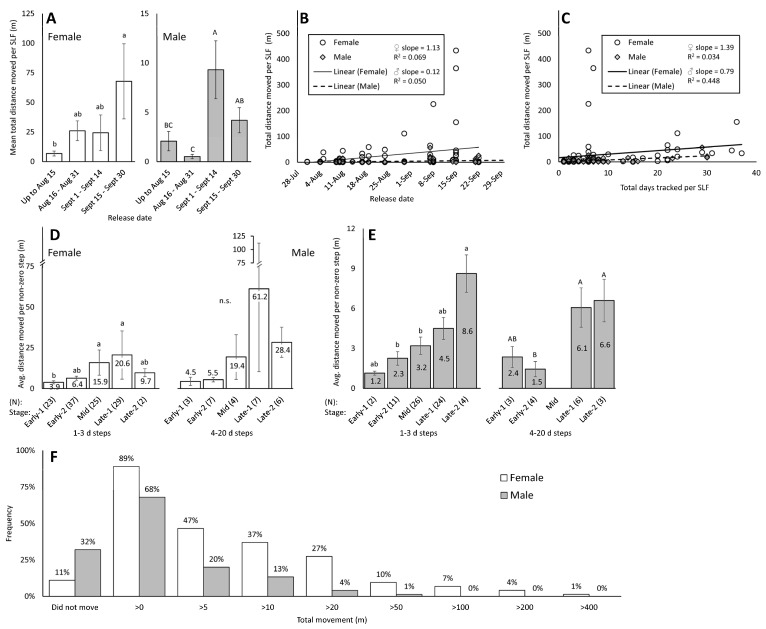
Parameters for distance moved by female (white) and male (gray) adult spotted lanternflies (SLF), *Lycorma delicatula,* tracked with RT (Experiment 1). Total distance moved (sum of all step-distances per SLF) by sex and release date are shown as means (±SE) (**A**) and individual SLF (**B**). The total time (days) that individual SLF were tracked is plotted against the total distance (m) they moved (with linear regression lines) in (**C**). The average non-zero distances travelled during steps that were 1–3 d in duration and 4–20 d in duration are shown for females (**D**) and males (**E**) over five adult stages, with the number of steps represented by each bar indicated by (N). The frequency of total distances that tracked individual SLF males and females traveled is shown in (**F**). In (**A**,**D**,**E**), bars in the same comparison with no letters in common are significantly different (ANOVA using ranked data for A, and log-transformed data for (**D**,**E**), followed by Tukey HSD means separations, *p* < 0.05, back-transformed data are shown).

To describe the average distance moved per insect, only the SLF that moved (the non-zero distances) were included in the following analyses. The mean of the total distance traveled by individual SLF that moved, tracked with RT, differed by release date (ANOVA on ranked data; females, *p* = 0.018, df = 3, 69, *F* = 3.565; males, *p* < 0.001, df = 3, 71, *F* = 10.288) (Figure 4A). The mean total distance moved by females increased gradually over the four release periods, whereas that of males was lowest for males released during late August and highest for males released during early September, corresponding roughly to Early-2 and Mid. The slope of the linear regression line for the relationship between distance traveled and release date in Figure 4B is positive and significantly different from zero for females (*p* = 0.025) but not for males (*p* = 0.053). Additionally, the slopes of the female and male regression lines were significantly different from each other (*p* = 0.042, df = 144, t-stat = 2.051). Analyses on total distance moved per SLF are complicated by having several individual SLF outliers that moved much longer distances (Figure 2D and Figure 4B) or that were tracked for much longer periods of time (Figure 4C). Using only the 1–3 d steps, it was revealed that average step distances of Mid and Late-1 females were significantly greater than those of Early-1 females (ANOVA on log-transformed data, *p* = 0.009, df = 4, 111, F-ratio = 3.58), peaking in Late-1 (Figure 4D). A similar pattern emerged with distances of steps that were 4–20 d long but with greater distances, greater variation, and no significant differences (*p* = 0.151, df = 4, 22, F-ratio = 1.88) (Figure 4D). For both 1–3 d and 4–20 d step durations, Late males traveled significantly longer distances than Early males (ANOVA on log-transformed 1–3 d steps, *p* = 0.008, df = 4, 62, F-ratio = 3.84; and 4–20 d steps, *p =* 0.022, df = 3, 12, F-ratio = 4.68), peaking in Late-2 (Figure 4E). In total, the majority of SLF males (80%) and females (53%) moved less than 5 m, and no males and 12% of females were recorded moving over 100 m (Figure 4F). The maximum total distance moved was 434 m by one of two females that traversed a small river, Jordan Creek, providing evidence that SLF can fly when tagged (Figure 1C, Table 1).

When the RT data for both years were combined for all males and females that moved, a significant difference in total distance moved per SLF was found between females (31.8 ± 9 m, N = 65) and males (6.3 ± 1.3 m, N = 51, *p* = 0.009, *t*-test) tracked with RT tags (Table 1). However, there was also a significant difference between the length of time females (8.8 ± 1.1 d, N = 73) and males (5.8 ± 0.8 d, N = 75, *p* = 0.034, *t*-test) were tracked with RT which confounds total distance.

#### 3.3.2. Rate of Movement of Adults with Radio Telemetry (Experiment 1)

**Figure 5:**
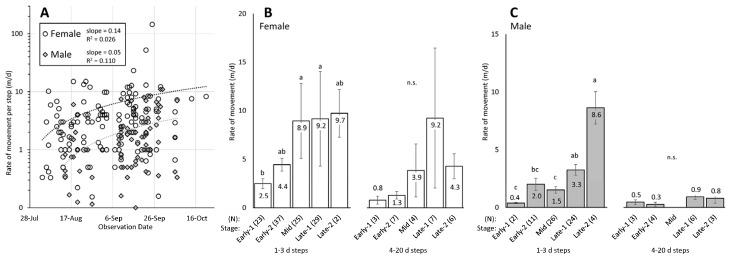
Rate of movement over time of adult female (white) and male (gray) spotted lanternflies (SLF), *Lycorma delicatula*, tracked with radio telemetry (Experiment 1). The movement rates (m/d) of all non-zero individual steps by date are shown in (**A**), plotted on a logarithmic scale. The mean rate of movement (m/d) (±SE) of SLF tracked over shorter tracking periods (1–3 d), and longer tracking periods (4–20 d) are shown for females (**B**) and males (**C**) over five stages. The number of steps represented in each bar is indicated by (N). Bars in the same comparison that do not share the same letter are significantly different (ANOVA on log-transformed data followed by Tukey HSD means separations, *p* < 0.05).

As with the maximum distance observed, the maximum rate of movement was accomplished by the same female, recovered on 27 September after moving 434 m from its previously known position three days prior, an average of 144.7 m/d. As with distance, a similar issue regarding outliers, changing frequency of movement, and differing amounts of time between observations (step duration) confound the calculation of the rate of movement. This is because if an insect was, for instance, only located after a period of 20 d, and had been sedentary for many days and then moved a long distance on one day, such behavior would not be captured by simply dividing distance by time. Therefore, the rate of movement per step was calculated using only the steps that were 1–3 d in duration, which included 183 of the 226 total steps that had movement. This revealed significant rates of movement differences between the five stages of adult females (ANOVA on log-transformed data; *p* = 0.006, df = 4, 111, F = 3.88) (Figure 5B), and males (*p* < 0.001, df = 4, 62, F = 9.18) (Figure 5C), similar to those of distance moved per step. The rate of movement using 1–3 d steps for females was significantly lower in Early-1 than in Mid and Late-1, which had similar rates of movement to each other. This is in contrast to males, which had a significant increase in rate of movement between Mid and Late. No differences over stages were found in the rates of movement of either sex with 4–20 d steps (females, *p* = 0.388, df = 4, 22, F = 1.08; males, *p* = 0.136, df = 3, 12, F = 2.24) (Figure 5B,C).

When the RT data were combined for all male and female steps with movement that occurred over a 1–3 d period, a significant difference in rate of movement was found between females (6.3 ± 1.5 m, N = 116) and males (2.6 ± 0.3 m, N = 67, *p* = 0.017, *t*-test) tracked with RT tags.

#### 3.3.3. Distance and Rate of Movement of Adults with Harmonic Radar (Experiment 2)

For SLF tracked with HR, there were only 23 non-zero-distance data points, with 9, 12, and 2 steps recorded during Early-1, Early-2, and Mid, respectively. The mean total distance moved per SLF did not vary between females (13.4 ± 6 m, N = 12) and males (11.2 ± 4 m, N = 8, *p* = 0.749, *t*-test) (Table 1). Likewise, for 1–3 d steps with movement, the mean rate of movement did not differ between females (3.5 ± 0.8 m/d) and males (3.3 ± 0.9, *p* = 0.848, *t*-test). Finally, the mean duration of tracking did not differ between females (7.5 ± 1.3 d, N = 19) and males (3.8 ± 1.5 d, N = 22, *p* = 0.078, *t*-test) tracked with HR. No differences were found between males and females, so they were combined for analysis over all stages and both years. For HR-tagged adults, as with RT-tagged adults (Figure 4B), the total distance moved per SLF appeared to increase depending on the release date, but was not significant (ANOVA on transformed data, *p* = 0.342, df = 2, 18, F = 1.139) (not shown). Although there was a positive trend between the total distance moved and release date, the linear relationship was not significant for the small number of HR-tagged SLF (R^2^ = 0.087, *p* = 0.193, slope = 1.03, N = 21). The rate of movement (m/d) (mean ± SE = 2.0 ± 0.3 m/d) of males and females combined did not vary significantly with step date (R^2^ = 0.104, *p* = 0.133, slope = −0.05, N = 23) not shown). The mean rate of movement for the 6 out of 23 non-zero-distance observations that occurred over a 1–3 d period was 3.4 ± 0.5 m/d, all occurring during Early-1. The observation that distance moved increased over time may have been confounded with the observation that the time between observations also increased over time for HR.

### 3.4. Direction

Turning angles for Experiments 1–3 are shown in Figure 2G,I,K. At least three different location observations are needed to calculate a turning angle, and this happened only twice in Experiment 2. Turning angles for both adults tracked with RT and nymphs tracked with HR were found to be random (Experiment 1: *p* = 0.578, Rayleigh test; *p* = 0.286, Hermans–Rasson test, Experiment 3: *p* = 0.782, Rayleigh test; *p* = 0.949, Hermans-Rasson test). While turning angles were shown to follow random distributions, the combined movement directions of tagged SLF in Experiment 1 were non-random (*p* = 0.037, Rayleigh test; *p* = 0.047, Hermans–Rasson test) (Figure 2H). However, movement directions for Experiments 2 and 3 were found to be random (Experiment 2: *p* = 0.455, Rayleigh test; *p* = 0.541, Hermans-Rasson test, Experiment 3: *p* = 0.651, Rayleigh test; *p* = 0.276, Hermans-Rasson test) (Figure 2J,L). When Experiment 1 data was split by year, the 2019 directions were correlated with movement against the mean prevailing wind direction (*p* = 0.040, V-test), while 2020 movement directions did not (*p* = 0.932, V-test).

### 3.5. Height

**Figure 6:**
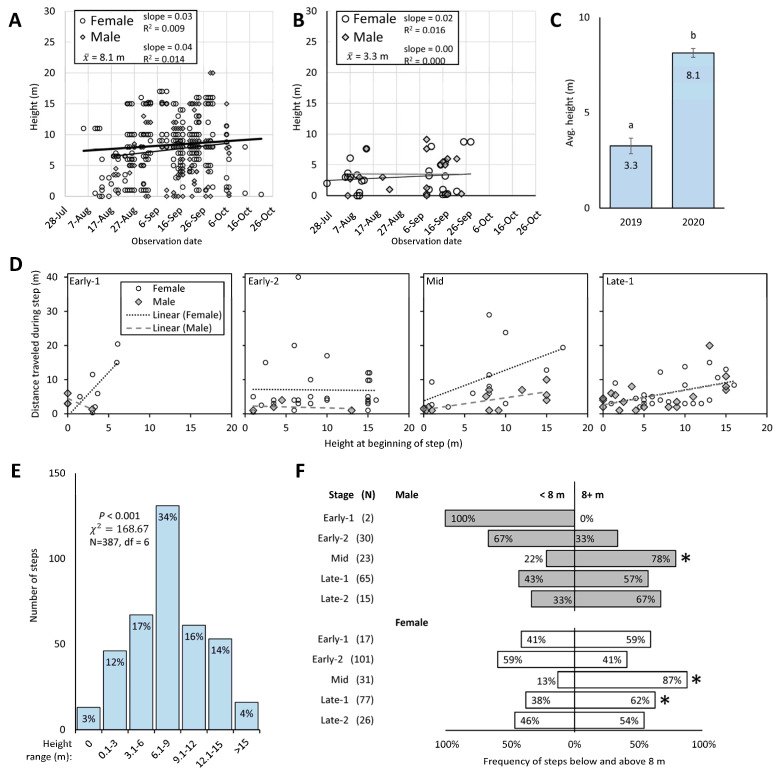
The height (m) above ground of male (gray diamonds) and female (open circles) (with linear lines for each) spotted lanternflies (SLF) *L. delicatula* tracked with radio telemetry (RT) is shown in scatter plots of raw step data by observation date in 2020 (**A**) and 2019 (**B**). Average SLF height in trees ± SE (m) at Trexler and Beaver Brook sites in 2019 and 2020, respectively (**C**) (Wilcoxon, *p* < 0.05, with different letters indicating significant difference). The number and frequency of SLF steps at different height ranges tracked with RT in 2020 for males and females combined over the entire season are shown in (**D**). The frequency of those steps that occurred above or below 8 m is shown in (**E**) for males (gray) and females (white) at each stage, with an asterisk indicating when the frequency was significantly greater in one height range than the other (Chi-square test, *p* < 0.05). Frequencies of male and female heights during each stage compared to the average height of 8 m are shown in (**F**).

Measuring the height above ground of tracked adult SLF resulted in the greatest number of data points from RT in 2020 (N = 388), followed by RT in 2019 (N = 48) (Figure 6A,B), HR in 2020 (N = 31), and HR in 2019 (N = 8) (not shown). The average height of the trees composing the contiguous forest canopy at the field site in 2020 (24.5 m) was 6 m higher than that in 2019 (18.6 m), thus the potential height for the SLF in 2019 was more restricted. As a result, SLF heights in 2019 ($\bar{x}$ = 3.3 ± 0.42 m) were significantly lower than in 2020 ($\bar{x}$ = 8.1 ± 0.23 m) (Wilcoxon test, *p* < 0.001, df = 1, χ^2^ = 42.6) (Figure 6C). On average in 2019 and 2020, respectively, SLF resided 15.3 m and 16.4 m below the average tree height. In the 2019 RT data, there were also long periods of time without data points, so our detailed analysis of SLF height is focused on the RT data from 2020.

Using sex and stage as factors, we tested the hypothesis that adults use height to gain distance, such that adults higher up in trees could launch themselves and fly farther. Using non-zero distance, 1–3 d steps, and excluding Late-2 for lack of data, we found that distance traveled was significantly affected by the height at the beginning of the step (factorial ANOVA on log-transformed distance; *p* < 0.001, F = 3.10, df = 15 [model], 98 [error]) with significant interactions between sex and stage (*p* = 0.041, F = 2.85, df = 3), and sex and height (*p* = 0.043, F = 4.19, df = 1), and significance for sex (*p* = 0.006, F = 7.91, df = 1). Linear regression analyses were conducted comparing the distance of non-zero, 1–3 d steps (again, excluding Late-2) to the height at the beginning of those steps, for males and females separately, and at each stage separately from Early-1 to Late-1 (Figure 6D). For females, distance traveled was significantly correlated to starting height during Early-1 (*p* = 0.034, F = 7.51, R^2^ = 0.556, slope = 2.6, N = 8) and Late-1 (*p* = 0.033, F = 5.21, R^2^ = 0.199, slope = 0.4, N = 23), with Mid approaching significance (*p* = 0.060, F = 4.39, R^2^ = 0.285, slope = 0.9, N = 13) and Early-2 having no significant relationship (*p* = 0.941, F = 0.01, R^2^ = 0.000, slope = −0.02, N = 32). For males, distance traveled was significantly correlated to starting height during Mid (*p* = 0.007, F = 11.07, R^2^ = 0.502, slope = 0.4, N = 13) and Late-1 (*p* = 0.026, F = 6.01, R^2^ = 0.273, slope = 0.4, N = 18), with no significant relationship during Early-1 (*p* = 0.434, F = 1.52, R^2^ = 0.603, slope = −1.1, N = 3) or Early-2 (*p* = 0.774, F = 0.11, R^2^ = 0.051, slope = −0.06, N = 4).

#### 3.5.1. Preferred Height of SLF

The frequency of tracked SLF that were found at different heights over the whole season (both sexes combined) varied significantly among 3 m height groupings (Pearson chi-square test, *p* < 0.001, χ^2^ = 168.67, df = 6, N = 387) (Figure 6E). There was a clear preference by SLF to reside in the 6–9 m height range. When heights of males and females were examined during each stage to see if they were found at the same frequency above and below their average height of 8 m, it was revealed that both males and females were found to be above 8 m significantly more than below 8 m during Mid (chi-square test; females, *p <* 0.001, G = 19.13, N = 31; males, *p <* 0.01, G = 7.80, N = 23) and this significance continued for females during Late-1 (*p* < 0.05, G = 4.74, N = 48) (Figure 6F). At all other stages, the frequency of males or females found above and below 8 m was not significantly different.

#### 3.5.2. Where Do Males Go during Early-2?

**Figure 7:**
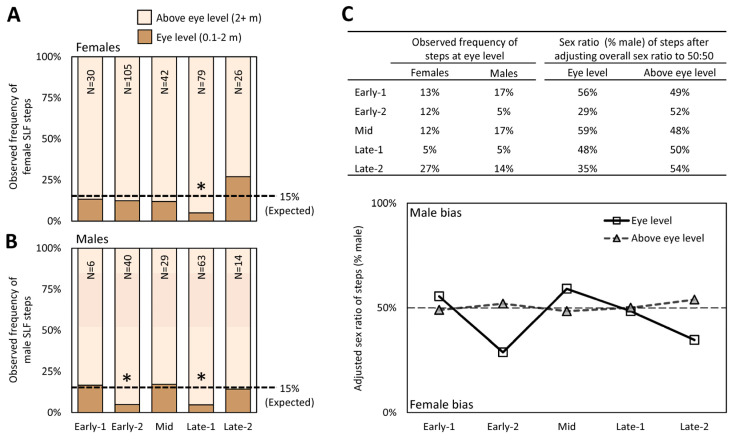
The step frequencies of female (**A**) and male (**B**) spotted lanternflies (SLF), *L. delicatula,* (including both RT- and HR-tagged SLF from both years) above and below eye level (<2 m) were compared against the expected frequency at eye level (15%), using a chi-square test for each stage. The total number of steps for each test is shown as N. Asterisks indicate that the frequency of steps above and below eye level deviated significantly from expected (*p* < 0.05). The frequencies that females and males were found at eye level for each stage were tabulated and used to calculate and plot the sex ratio of steps at eye level (solid line with squares) and above eye level (dashed line with triangles) (**C**).

We also tested the hypothesis that the perceived sex ratio shift, when males are perceived to become scarce during Early-2, was due to males moving higher than eye level (2 m) at a greater pace than females during that stage. Since 15% of all steps occurred below 3 m and the remaining 85% were above that (Figure 6E), a chi-square test (α = 0.05) was used to determine if the frequency of all male and female steps during each stage deviated from these expected frequencies. We found three instances when the frequencies significantly deviated from expected. During Late-1, the frequencies of both female steps (chi-square test, G = 7.90, N = 79; Figure 7A) and male steps (G = 6.76, N = 63; Figure 7B) occurred above eye level 95% of the time, significantly more than the expected 85%. However, during Early-1, only male steps occurred above eye level at a significantly higher frequency than expected (95%, G = 4.06, N = 40; Figure 7B). The frequencies of all tracked steps that were found at eye level or above eye level for each sex were used to calculate the perceived sex ratios at eye level and above eye level, assuming an overall sex ratio of 50:50, and these were plotted over time (Figure 7C). The resulting graph shows that at eye level, a female skew in sex ratio (29% male) would be perceived at Early-2, then subsequently skew slightly male during Mid before gradually returning to a female skew again in Late-2, all while the overall population sex ratio remains at 50:50 (Figure 7C). Although not significant, the frequency of female steps at eye level was greatest (27%) during Late-2 (Figure 7A).

### 3.6. Host Plant Associations

**Figure 8:**
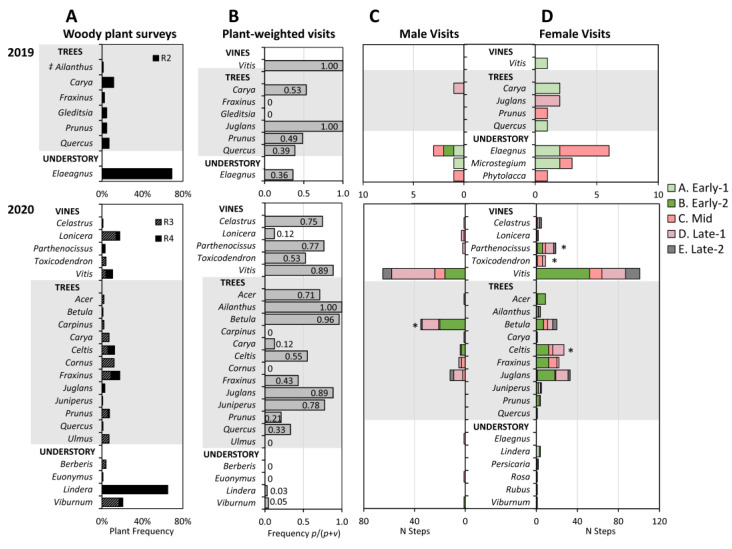
Survey results (**A**) showing the frequency of woody plants by genus in a 15 m radius around the release trees at the three main release sites for 2019 (R_2_) and 2020 (R_3_, and R_4_). The only *Ailanthus* present in either survey was the release tree in 2019 (‡), and SLF that never left their release trees were excluded from this analysis. The plant-weighted visit frequency (omitting release trees) is shown by genus in (**B**), where *p* is the plant frequency and *v* is the frequency of visits by spotted lanternflies (SLF), *L. delicatula*. A result between 0 and 1 is given, where 0.5 indicates plants were visited at the same frequency as their presence, greater than 0.5 suggests that species was favored, and less than 0.5 suggests that species was avoided. The number of encounters (N steps) with host plants recorded for adult male (**C**) and female (**D**) SLF, tracked with radio telemetry in 2019 (top) and 2020 (bottom) is shown by genus, with stages indicated by different colors. Asterisks indicate when one sex was found on a species at a relative frequency significantly greater than the other sex (chi-square test, *p <* 0.05).

The number of host associations recorded for adult RT-tagged SLF in 2019 and 2020, were 48 and 391, respectively. Of those, 48% and 62% were found on host plants other than their release trees in the two respective years, with a higher proportion staying on *A. altissima* release trees in 2019. In 2020, 16% of observations involved SLF that returned to the release tree after being found elsewhere. The plant survey at the 2019 site (R_2_) turned up nine woody species, six of which were visited by SLF (excluding the *A. altissima* release trees) in addition to four additional herbaceous and woody species that were visited but not on the survey. The survey of 2020 sites (R_3_ and R_4_) found 25 woody species, 16 of which were visited by SLF in addition to 8 other herbaceous and woody species that were visited but not on the survey (Figure 8A–D). In 2019, SLF were predominantly found on autumn olive *Elaeagnus umbellata* Thunb. (Rosales: Elaeagnaceae), the most abundant species, whereas in 2020, SLF were predominantly found associated with wild grape, *Vitis* sp., growing epiphytically on trees, or associated with the trees themselves, and were not frequently associated with understory species (Figure 8C). A detailed description of the woody species with which SLF associated follows.

#### 3.6.1. Associations with Individual and Intertwined Plant Species

Five species of vines were observed being visited in combination with other plants or by themselves, and consisted of *Vitis*, Oriental bittersweet *Celastrus orbiculatus* Thunb. (Celastrales: Celastraceae), Japanese honeysuckle, *Lonicera japonica* Thunb. (Dipsacales: Caprifoliaceae), Virginia creeper *Parthenocissus quinquefolia* (L.) Planch. (Vitales: Vitaceae), and poison ivy *Toxicodendron radicans* (L.) Kuntze (Sapindales: Anacardiaceae). For this analysis, which included steps on release trees, each of the intertwined species received a step count when it was unclear which of the intertwined species the SLF was on. Five plant species in 2020 stood out for their much higher number of visits than other species: *Vitis*, black walnut *Juglans nigra* L. (Fagales: Juglandaceae), sweet birch *Betula lenta* L. (Fagales: Betulaceae), *Fraxinus* spp. (Lamiales: Oleaceae) (including *F. americana* L. and *F. pennsylvanica* Marshall) and common hackberry *Celtis occidentalis* L. (Rosales: Cannabaceae). The most frequently seen plant associations, including when multiple species intertwined, were *B. lenta* + *Vitis* (89 steps), *J. nigra* + *Vitis* (72 steps), *Vitis* (49 steps), *J. nigra* (32 steps), *B. lenta* (21 steps), *Fraxinus* spp. (21 steps), *C. occidentalis* + *Vitis* (18 steps), *C. occidentalis* (11 steps), and *C. ovata* (9 steps). Some plant associations in 2020 were only observed when intertwined with more commonly visited *Vitis* or *J. nigra*, these were: one record of boxelder *Acer negundo* L. (Sapindales: Sapindaceae), six records of C. *orbiculatus*, two records of mile-a-minute *Persicaria perfoliata* (L.) H. Gross (Caryophyllales: Polygonaceae), and four records of black cherry *Prunus serotina* Ehrh. (Rosales: Rosaceae).

#### 3.6.2. Woody Plant Survey and Plant-Weighted Visits

The total frequency of woody plants surveyed in a 15 m radius around the main release trees in 2019 and 2020, can be seen by genus in Figure 8A. The plant-weighted visit parameter can range from 0 to 1, where plants that were visited at the same frequency as their occurrence received a score of 0.5, scores of less than 0.5 indicate that they were visited less often than their occurrence would predict (avoided or ignored), and scores greater than 0.5 indicate they were visited more often than their occurrence would predict (favored) (Figure 8B). Some differences in host associations between the two years and locations were clearly due to differences in available flora at the different field sites.

In 2019, the main host associations were with the *A. altissima* release trees (not shown), and *E. umbellata*, which was the most abundant woody plant (Figure 8). When considering plants in the 15 m radius, shagbark hickory *C. ovata* and *P. serotina* had scores near 0.5. The remaining two species with scores over 0.5 were *J. nigra*, and *Vitis* sp., both of which were not found within the 15 m radius so received scores of 1.0.

In 2020, the only *A. altissima* present were more than 15 m away from release trees, but a few females located them, so *A. altissima* was the only species with a score of 1.0 that year. Other species in 2020 with scores higher than 0.8 were *B. lenta*, *J. nigra,* and *Vitis.* Species in 2020 with scores between 0.7 and 0.8 were *C. orbiculatus, P. quinquefolia*, *Acer* spp. (which included *A. negundo,* Norway maple *A. platanoides* L., and red maple *A. rubrum* L.), and Eastern red cedar *Juniperus virginiana* L. (Cupressales: Cupressaceae).

The remaining plant species that were visited but at lower frequencies were chestnut oak *Quercus montana* Willd. (Fagales: Fagaceae), Japanese stiltgrass *Microstegium vimineum* (Trin.) A. Camus (Poales: Poaceae), and pokeweed *Phytolacca americana* L. (Caryophyllales: Phytolaccaceae) in 2019, and *E. umbellata*, white oak *Quercus alba* L. (Fagales: Fagaceae), multiflora rose *Rosa multiflora* Thunb. (Rosales: Rosaceae), spicebush *Lindera benzoin* L. (Laurales: Lauraceae), black raspberry *Rubus occidentalis* L. (Rosales: Rosaceae), and *Viburnum prunifolium* L. (Dipsacales: Adoxaceae) in 2020.

Several species found in the woody plant surveys were not observed being visited by SLF. In 2019 these included honey locust *Gleditsia triacanthos* L. (Fabales: Fabaceae), northern red oak *Quercus rubra* L. (Fagales: Fagaceae), *Fraxinus* sp., and pignut hickory *Carya glabra* Miller (Fagales: Juglandaceae). In 2020 these included sugar maple *Acer saccharum* Marshall (Sapindales: Sapindaceae), Japanese barberry *Berberis thunbergii* DC. (Ranunculales: Berberidaceae), American hornbeam *Carpinus caroliniana* Walter (Fagales: Betulaceae), bitternut hickory *Carya cordiformis* (Wangenh.) K. Koch (Fagales: Juglandaceae) American dogwood *Cornus florida* L. (Cornales: Cornaceae), burning bush *Euonymus alatus* (Thunb.) Seibold (Celastrales: Celastraceae), Amur honeysuckle *Lonicera maackii* (Rupr.) Maxim. (Dipsacales: Caprifoliaceae), *Q. rubra,* pin cherry *Prunus pensylvanica* L.f. (Rosales: Rosaceae), and slippery elm *Ulmus rubra* Muhl. (Rosales: Ulmaceae).

#### 3.6.3. Sexual Differences in Host Plant Associations

The total number of steps observed on each plant during each stage by males and by females are shown by genus in Figure 8C,D, respectively. The frequency at which each sex visited plants was compared to see if there were sexual differences in plant preference. One species, *B. lenta*, had a significantly higher relative proportion of male visits than female visits (*p* < 0.001, G = 23.10, df = 2, N = 68) (Figure 8C). Three plants that had a significantly higher relative proportion of female visits than male visits were: *C. occidentalis* (chi-square test, *p* = 0.006, G = 7.85, df = 2, N = 26)*, T. radicans* (*p* = 0.010, G = 9.28, df = 2, N = 7), and *P. quinquefolia* (*p* = 0.007, G = 7.74, df = 2, N = 17) (Figure 8D). The 2019 data lacked enough steps for statistical analysis.

### 3.7. SLF Density and Orientation

**Figure 9:**
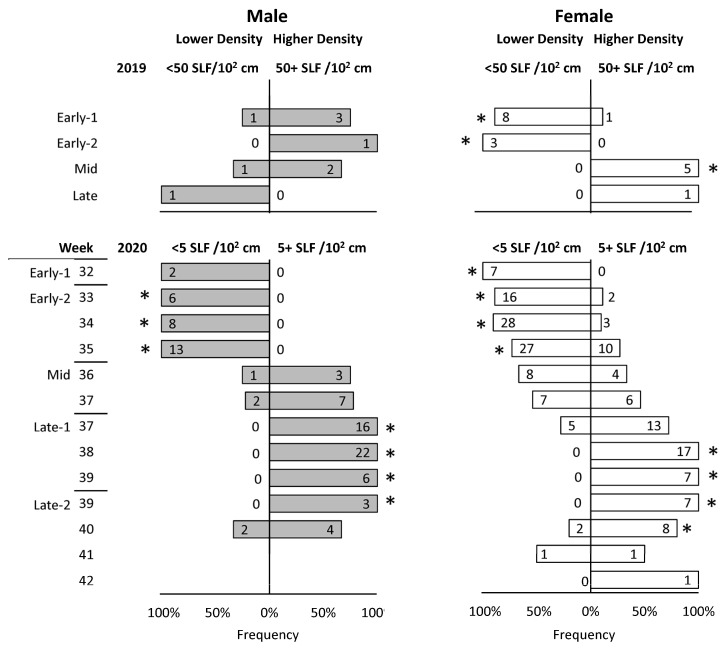
The frequencies at which adult spotted lanternfly (SLF), *L. delicatula,* males (gray) and females (white), tracked with both technologies in 2019 and 2020, and excluding those that never left their release trees, were found on the same surface as naturally occurring SLF populations at lower or higher densities (as estimated at eye level), at different stages. Asterisks indicate when SLF were found near one density significantly more than the other (*p* < 0.05, chi-square test). Numbers inside bars represent numbers of SLF found.

The observed density of SLF on surfaces where tagged SLF were located was recorded for 122 and 480 steps in 2019 and 2020, respectively. However, after excluding steps when SLF were found dead, or only the detached tag was found, or SLF remained on the tree on which they were released, only 27 and 276 steps in 2019 and 2020, respectively, were used for analysis. The density of the naturally occurring SLF population, as estimated at eye level, varied throughout the season and by year. The population density at the 2019 field site was much higher than at the 2020 field site. In the 2019 field season, roughly half of the steps of tracked SLF were located on surfaces where more than 50 SLF were naturally occurring in the most densely populated 10 × 10 cm^2^ area of the tree trunk at eye level, whereas in the 2020 field season, roughly half of all steps occurred on surfaces shared by a density of more than 5 SLF per 10 × 10 cm^2^ area. Using these cutoffs for 2019 and 2020, chi-square tests were conducted on each sex and time interval to determine if tracked SLF showed any density preferences over time. Because there were fewer data points in 2019, stages were used as time intervals, but in 2020, stages and weeks were used as time intervals (in some weeks, the first half of the week was in one stage and the second half was in another stage). It was revealed that both males and females were preferentially associated with lower densities during Early-1 and Early-2. However, a shift in density preference began in both sexes at Mid, with males completing the shift half a week earlier than females during Late-1 (Figure 9). In Late-2 males began to shift back, followed by females half a week later. During all of Late-1 and half of the first week of Late-2, males were found exclusively associated with the higher SLF densities on plants, and females followed the same pattern but delayed a few days. 

### 3.8. Habitat Choice

Habitat types were characterized as field, forest edge, or inner forest, with forest edge defined as in a forest but within view of a field. In most cases, SLF were found in the same habitat where they were released. The only exception was a female SLF released at R_2_, an *A. altissima* tree at the forest edge in 2019, which was found more often in the inner forest than the forest edge or field. The type of habitat in which tracked SLF were located (excluding SLF that never left their release trees) appeared to vary depending on the release point. Most SLF released from an *A. altissima* tree isolated in a field (R_1_), a *J. nigra* tree in the inner forest (R_4_), and a *J. nigra* on a forest edge (R_5_), did not leave the habitat type where they were released (Table 2). Most of the movement out of the release habitat and into other habitats occurred from R_2_ which was an *A. altissima* tree on the forest edge, and R_3_ which consisted of a *C. ovata* tree and a *B. lenta* tree in the inner forest. For those that left the forest edge where they were released (R_2_), a greater proportion of males and females were found in the inner forest than in the field. Those that left their release habitat in the inner forest (R_3_ and R_4_) were also more frequently found at the forest edge than in the field.

### 3.9. Radio Telemetry and Harmonic Radar Performance

#### 3.9.1. Impacts of Technology on Tracking Adult SLF Movements

**Figure 10:**
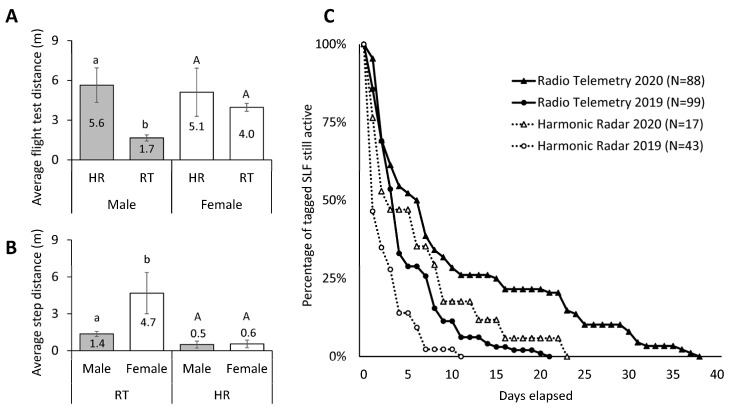
Comparison between (**A**) radio telemetry (RT) and harmonic radar (HR) showing the average distances (±SE) flown during flight tests by males (gray) and females (white) affixed with either HR or RT tags (Wilcoxon test comparing tag type within each sex); (**B**) non-zero 1–3 d step distances of males and females for each tag technology (ANOVA on log-transformed data); and (**C**) the amount of time over which tracking took place for individual *L. delicatula*, spotted lanternflies (SLF), until the last time each SLF was located. In statistical comparisons, bars with different letters are significantly different.

With RT tags being heavier than HR tags, and females being larger than males, we tested whether tag type affected the flight capabilities of males and females. The average distances that male and female SLFs flew during flight tests, including zeros, are shown in Figure 10A. The type of tag had a significant effect on distance flown by males in flight tests (Wilcoxon test, $\chi^{2}$ = 11.05, *p* < 0.001, df = 1, N = 7, 43), but not females ($\chi^{2}$ = 0.102, *p* = 0.749, df = 1, N = 9, 39). Males affixed with the heavier RT tags flew an average of 1.7 (±0.2) m (N = 43), as compared to females with RT tags, which flew 4.0 (±0.3) m (N = 39). Males with the lighter HR tags flew 5.6 ± 1.3 m (N = 7) in flight tests, similar to females with HR tags which flew 5.1 ±1.8 m (N = 9). Thus, in flight tests the males with HR tags were able to fly roughly 3.3 times farther than males with RT tags. A similar pattern was seen when all non-zero 1–3 d step distances by males were compared to those by females affixed with RT (ANOVA on log-transformed data, F = 11.52, *p <* 0.001, df = 1, 181) or HR tags (F = 0.18, *p =* 0.692, df = 1, 4) (Figure 10B).

The rate of locating (ability to find) tagged SLF for Experiment 1 (RT of adults) and Experiment 2 (HR of adults) is graphed in Figure 10C. When looking at all the adult SLFs tagged and released, both RT and HR had improved rates of location and longer tracking times in 2020 compared to 2019, with RT having a higher location rate overall. The longest time we were able to locate live SLF with HR tags was 22 d after their release, as opposed to 37 d using RT (Figure 10; Table 1). However, average tracking time did not differ significantly between RT and HR (Wilcoxon test, $\chi^{2}$= 0.03, *p* = 0.862, df = 1, N = 108, 23).

The recovery rate, number of observations per insect, rate of movement per SLF, and rate of movement per female SLF were significantly lower for HR than for RT (2-tailed *t*-test, *p* < 0.05), with remaining parameters (time tracked per SLF, total distance, per SLF, female, and male, and rate per male) not differing significantly between tracking technologies (Table 1). 

#### 3.9.2. Adult Movement and Harmonic Radar Antenna Material

SLF adults were successfully tracked with HR in both 2019 at the Trexler Nature Preserve using platinum wire tags, and in 2020 at Beaver Brook Wildlife Management Area using nitinol wire tags. When separating the movement parameters by antenna material (year), nitinol tag values were numerically, higher than those for platinum tags in every parameter compared in Table 3 except for maximum total distance and mean rate moved. The mean number of observations per SLF was significantly higher with nitinol tags in 2020 than with platinum tags in 2019 (Table 3).

#### 3.9.3. Nymph Movement and Harmonic Radar Antenna Material

Step frequency and distances, total distance moved, and directionality of movements of fourth-instar SLF nymphs tracked with HR (Experiment 3) can be found in Figure 2C,F,K,L. As with adults, the most common step distance for nymphs was zero m (Figure 2C), but interestingly, nymphs in the corn field were always found at new locations with all the zero step-distances shown in Figure 2C attributable to the forest edge sub-experiment. Fourth-instar nymph movements were successfully tracked up to 5 days after release with a maximum distance moved of 27.6 m (Table 4 and Table 5). All tagged SLF nymphs were visually observed at all time points in the corn field, while several tagged nymphs at the forest edge ascended trees and so were only detectable by HR signal.

Differences in movement parameters were found both between the types of tags used (nitinol vs. tungsten) and between the tracking locations (within the corn field vs. at the field/forest interface). When looking at the movement data by tag type and by location, nitinol values were numerically higher than those for tungsten in every comparison except mean distance moved per day within the cornfield. Additionally, mean time tracked in the corn (Table 4) and mean total distance at the field/forest interface (Table 5) were significantly higher for nitinol.

**Figure 11:**
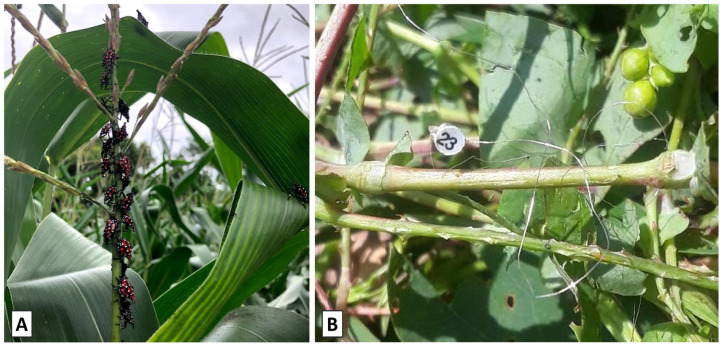
Fourth-instar nymphs of spotted lanternfly, *L. delicatula*, observed feeding on corn in 2019 in Lehigh County, PA, USA (**A**). The tangling of an HR tag made with a Wollaston process (platinum) wire antenna effectively reduced antenna length and severely attenuated the detection range of tags (**B**).

All movement parameters were numerically higher in the corn field (Table 4) than at the field/forest interface (Table 5) with the exception of mean number of observations per SLF. When tag types are compared across both field locations combined, the mean number of observations per released SLF was higher for nitinol (3.2 ± 0.2) than for tungsten (2 ± 0.3, *p* < 0.01, *t*-test) while total distance moved, total time tracked, and distance tracked were all numerically higher for nitinol. When movement parameters were compared between field locations, combining tag types, total distance moved, total time tracked, and distance tracked were all significantly higher in the corn field, while the number of observations per SLF did not differ between the locations (Table 6). Corn is not thought to be a preferred host for SLF but we observed naturally occurring nymphs feeding and honeydew accumulating on corn both in 2019 and 2020 (Figure 11A).

## 4. Discussion

### 4.1. Benefits and Shortcomings of Radio Telemetry and Harmonic Radar to Track SLF

In the current study, we successfully tracked SLF adults using both HR and RT. When comparing these two technologies for use tracking SLF movements, each revealed several advantages and disadvantages. For adults, tracking with RT produced the most data (Figure 1A), for several key reasons discussed below.

#### 4.1.1. Attenuation of Signals When Tracking Long-Distance Steps

Overall adult recovery rates were significantly lower with HR (55%) than with RT (80%) (Table 1). One possible reason is that SLF may have been traveling outside of the HR detection range. The maximum distances traveled by tracked SLF adults in 2019 and 2020, respectively, were 60 and 50 m with HR, and 434 m and 156 m with RT, which corresponds with the respective maximum distances that HR and RT signals can be detected. Although the two technologies did not differ statistically in the distance parameter, RT appeared to perform better for tracking SLF movements over longer distances.

SLF adults could be tracked more efficiently and over a longer time and distance with active RT tags than with passive HR tags (Figure 10, Table 1). Regardless, most tagged SLF were tracked only for the first few days before tracking ended due to death or loss, with far fewer tagged SLF tracked longer than seven days. However, the information generated by the few insects that were tracked over longer periods was of great informative value.

The higher recovery rates and longer tracking times were mainly due to the stronger signals of RT tags resulting in greater detection distances. The HR signal attenuated after a shorter distance, and therefore, SLF that traveled distances over 50 m were out of range of HR, whereas RT-tagged SLF could still be detected after traveling a similar distance. As a result, only the HR-tagged SLF with the shortest movements were likely to be tracked, and those that moved farther were filtered out, giving what is likely a false impression that HR-tracked SLF had less movement.

#### 4.1.2. The Importance of a Unique Signal

In order to record the position of a specific insect being tracked, and associate that position with its previous position, the identity of the insect carrying the tag had to be confirmed. When locating a RT tag, even if the insect was high in the tree and unable to be seen, the signal produced by each tag was unique, so the location of the unique signal could be attributed to the specific insect wearing that tag and associated with its previous positions, even though the insect was obscured from view. However, if too many RT tags were activated, the RT tags could simultaneously emit signals rendering them undecipherable, so we were limited to using about 8 tags in one place and had to carefully time their activation in order to space out when signals were emitted. Conversely, because HR tags do not convey any unique information through their reflected signal, even when the signal from an HR tag was located, its position was not able to be recorded unless its numbered tag or marked wings could be seen, or it was the only HR-tag released in the area, because it could not be attributed to an individual SLF. This resulted in fewer HR tracking points and more lost HR-tagged SLF. Although HR tags could often be detected without seeing them, in those cases it was often impossible to determine which SLF was being detected because the HR signal is merely reflected and is not unique. Attempts to rectify this problem included limiting the number of insects released and tracking with HR tags in a given area in order to allow HR-tracked insects to be identified without being seen. This also contributed to the reduced data points for HR tags.

#### 4.1.3. Distortions Due to Tag Technology and Sex of Adult SLF

Tag mass as a proportion of SLF body mass, sexual differences in body mass, differences in tag detection range, and the added difficulty of locating SLF with longer movement bouts, combined, may have distorted the results. Tag mass and size [30,54] have presented obstacles for tracking tagged insects. Tags are required to be extremely small and light when used with insects. An often-used benchmark is that the tag mass should be less than 5% of the insect mass, although the empirical basis for this limit is weak [30]. For RT-tagged SLF, tags ranged from roughly 30–60% of body mass. While this is well above the 5% benchmark, pre-release testing showed that RT-tagged SLF could fly, and anecdotal observations, such as tagged SLF crossing a small river in the study site (Figure 1C), suggest that at least some tagged SLF continued to make flights throughout the tracking period. For HR-tagged SLF, the range was roughly 3–6% tag-to-body-weight. For adult female SLF, a body mass of 200–300 mg is associated with longer flight distances while body masses of over 400 mg are associated with shorter flights [55]. As female body masses in particular increased throughout the season, it is reasonable to assume that movement distances were more negatively affected by tag mass.

Even given some of the long-range SLF movements detected in this study (Figure 1 and Figure 2D), as the distance moved increased, so did the difficulty associated with locating tagged SLF. For every meter further an SLF moves, the search area needed to locate the tagged insect increased by the power term

π × *d*^2^ (2)

where *d* is the distance moved. For an SLF that moves 10 m (the average step distance observed for RT-tagged SLF), the search area is roughly 314 m^2^, while for an SLF that moves 100 m the search area increases to almost 31,500 m^2^. Both the changing tag-to-body mass ratio and the increasing search area size associated with longer movements likely contribute to this study not detecting significantly increasing movement distances as the season progressed.

Recorded distances of each sex were affected unevenly by tag technology as a result of one sex being larger than the other and one tag being heavier than the other. Although males and females with light-weight HR tags performed similarly in flight-tests, males with HR tags were capable of flying significantly farther than males with RT tags, while females in flight tests were not significantly affected by the added weight. A similar significant disparity was observed in the step distances of males and females carrying these tags in the field (Figure 10A,B). Since flight tests found that female movement was not significantly affected by the tags, females that moved long distances were likely able to be tracked farther with RT tags, but those with HR tags likely traveled out of detection range and were lost more often. It is also important to note that if HR-tagged males that traveled far were filtered out due to signal attenuation, and RT-tagged males were unable to move as far due to tag weight, one could incorrectly interpret the resulting data to mean that males moved less than females, but for the above-stated reasons such a conclusion cannot be drawn based on this data. However, evidence that males may not travel as far as females has been documented elsewhere [56].

#### 4.1.4. Harmonic Radar Antenna Material

Tag entanglement [57], and antenna deformation [54] are both obstacles in tracking insects. Of several materials tested for the HR antenna affixed to the insect, Wollaston process wire, which is a platinum wire clad in silver, is light and flexible but quickly became deformed by bending and tangling, thus reducing the detection distance of the tags and encumbering movement (Figure 11B). Tungsten wire, on the other hand, was inflexible and likely restricted the movement of the SLF nymphs. Additionally, the tags made with tungsten were heavier than those made with nitinol or Wollaston process wire. Superelastic nitinol wire (Figure 2B,C) produced superior results. Importantly, it was both lightweight and flexible, and allowed for unimpeded insect movement while retaining shape and length in order to efficiently reflect the signal and maximize detection range. In particular, the tracking of nymphs succeeded due to the use of superelastic nitinol wire as the HR tag antenna. Other metals such as copper [47], copper-cladded steel [46], silver-plated copper [39], steel [39], and aluminum [45,48] have been used as antennas in previous studies but have resulted in wire deformation [46,58] and entanglement or encumbrance [39,48]. Nitinol was found to be an ideal material for HR tags as it is lightweight, relatively inexpensive, and possesses the necessary conductive properties. The superelasticity of nitinol allows it to undergo substantial deformations before returning to its original shape [59]. The advantages of nitinol were clearly demonstrated when compared to the other two metal wires used in this study.

#### 4.1.5. Economic and Design Considerations of Tags

There was a considerable difference in cost between the two tag technologies. At the time these studies were conducted, the single-use RT tags, which had a 3-month shelf life, cost approximately $285 per tag. Conversely, each reusable HR tag could be constructed for under $10 and had an indefinite shelf-life. In addition, the fact that RT tags are battery-powered meant that upon activation, they had a finite lifetime in the field that was inversely related to how often they broadcast their signal, which could be adjusted. With our settings, each tag was not expected to exceed 20 days of battery life (although some exceeded that expectation). In addition, attaching the RT tags was more difficult due to their larger size, rounded shape, and heavier weight of approximately 150 mg. Therefore, more experimentation and practice were required to establish the best attachment method without harming or impeding the insect. HR tags had the advantage of being lighter (approximately 15 mg), easier to attach, and with no battery, they could theoretically function indefinitely in the field.

Recovery rates and durations improved from 2019 to 2020 due to design improvements in both types of tags. The RT tags used in 2019 were the first version of a new model of tag that was the smallest available at the time, and our study was the first to test the new product, thus an appreciable number of tag malfunctions initially occurred resulting in lost tags (and SLF) that year. With subsequent improvements from the manufacturer, few if any RT tags malfunctioned in the second batch received in 2019, and in the following year in 2020. The HR tags also changed in 2019 compared to 2020, when fine Wollaston wire antennae were replaced with flexible and sturdier nitinol wire antennae. The improved RT and HR recovery rates from 2019 to 2020 are attributable in large part to the improvements in tag designs for both types of tags. As both technologies continue to improve, so will our ability to track insects.

#### 4.1.6. Overall Performance of Tag Technology for Tracking SLF

For SLF adults, particularly females, the advantages of RT outweighed the disadvantages due to the tendency of some adults to move long distances at once, which quickly put them out of HR range, and they were lost. Thus, more complete movement data was obtained using the RT tracking method when compared to HR. Although males wearing RT tags could not move as far due to tag weight, males wearing HR tags could move out of range, thus both technologies performed poorly at tracking male long-distance movements. The distance adult males moved was significantly impacted by the larger RT tags, so although RT tags allowed for better tracking of males than HR tags, we did not get as complete a picture of adult male potential travel distances as we did with females.

The usefulness of HR was well-demonstrated with the smaller SLF nymphs which could not carry a RT tag due to their size and weight, and which never traveled outside of the range of the HR tags during our experiments. Currently, radio telemetry tags are not available in sizes small enough to use for tracking SLF nymphs, so tracking of nymphs must be conducted using harmonic radar. Overall, the choice of tracking method will depend on the specific needs and constraints of the insect being studied.

### 4.2. Tracking SLF Nymphs with Harmonic Radar

While close to 100 species of terrestrial arthropods have been studied with tracking devices [30], the only previous study of an immature insect was accomplished by Kirkpatrick, et al. [60] which used HR to track *H. halys* nymphs in both host (apple tree) and non-host (mowed grass) vegetation. There has also been previous work with SLF nymphs by Jung et al. [42] that investigated potential negative effects of attaching HR tags to four instars of SLF nymphs and found that the HR tags did not significantly impair either jumping ability or survivorship. While the tags used by Jung et al. [42] differ from those used in the current study, both in size and mass, both studies show minimal impacts of tag attachment. When looking at subsequent location observations, the most common outcome was that fourth-instar nymphs remained in the same location. Of those that moved, nymph turning angles were random, perhaps showing that nymphs either don’t orient to vertical structures as adult SLF do or were unable to, given the field conditions of this study. Nymphs moved further in cornfields, by both mean and maximum distances along with mean rate, when compared to the forest edge. This may suggest that nymphs in corn were attempting to reach a more suitable habitat or food source and therefore moved further. In at least three cases, nymphs moved to *A. altissima* and then did not move further.

### 4.3. Adult SLF Behavioral Changes over Time

SLF spend much of their 10–16-week adulthood feeding, with specific periods of time marked by behavioral changes in host preference, sex ratio, aggregation, courtship, mating, dispersal, and oviposition [15,16]. This study quantifies and correlates behaviors of adult SLF tracked in the field in defined stages and improves our understanding of the frequency and distance of their movements, heights at which they reside, the relationship between height and subsequent distance moved, aggregation and courtship behavior, host preferences, and the phenology thereof. Previously, phenological studies of SLF activities characterized the physiological states of SLF adults as Early-1, Early-2, Mid, Late-1, Late-2, and Late-3 (described above) [15]. While summarizing when tracked SLF were primarily engaged in certain activities, it became clear that the majority of these behaviors either began during Mid, after mating was first observed, or began or intensified during Late-1 after oviposition was first observed, about 1–2 weeks later (Table 7). The following discussion underscores specific related behaviors followed by a phenological overview.

#### 4.3.1. Aggregation and Peak Mating Time

Aggregation behavior was previously demonstrated in the field for all SLF nymphal stages [61], and adult females at all stages [15], whereas males in the latter study oriented to higher-density trees baited with caged females or males only after mating commenced. Thus, adult males orient to, and form, tight aggregations with females for courtship and mating, and are guided at least in part by semiochemicals [62,63]. During courtship, males and females huddle in tight clusters, unlike looser aggregations seen before Mid [15]. In this study we quantified, over time, the frequency of tracked adults that oriented to trees with naturally occurring SLF quantified as being densely or sparsely assembled. By conducting chi-square tests at each time interval, the time during which SLF orientation shifted from significantly choosing sparse assemblies to significantly choosing dense assemblies likely indicated when most mating activities occurred (Figure 9, Table 7). All male and most female SLF in Early-1 and Early-2 oriented to trees with less dense or no aggregations, but during Mid, males shifted their behavior and more frequently oriented to trees with dense assemblies, and females started to make this transition as well. For males, the shift was complete in the first week of Late-1 when 100% of males oriented to trees with large tight SLF clusters, with females completing the shift a few days later, after which 100% of both sexes oriented to trees with dense aggregations for the next two weeks. In 2020 this peak aggregation occurred during the latter half of week 37, and all of weeks 38 and 39. Although fewer data points were gathered in weeks 40–42, Late-2, and the beginning of Late-3, fewer of the trees visited by tracked SLF at that time had dense aggregations as courtship activities were followed by oviposition.

Based on the timing of aggregation behavior which transitioned during Mid and climaxed during Late-1 (Figure 9), as well as corresponding changes in height, the data suggests that Mid is a transitional period when some SLF begin mating, and that peak mating time occurs during Late-1 as the first mated females descend and oviposit. Peak aggregation lasted 2.5 weeks encompassing all of Late-1 and a few days in Late-2, and was flanked by periods of less intense aggregation behavior in Mid and Late-2.

#### 4.3.2. Height, Mating, and Dispersal

Nearly 90% of steps took place above eye level and out of sight for an average observer on the ground. SLF resided on average 15–16 m below the treetops in both years. In 2020 at Beaver Brook, where trees were on average 24.5 m tall, both sexes were found most often between 6–9 m high in trees with the average height being 8 m. However, during Mid, significantly more than half of male (78%) and female (87%) steps occurred above 8 m, as well as female steps during Late-1 (62%), corresponding to when mating begins and peaks, indicating that most aggregation, courtship, and mating occurs in the canopy.

SLF are weak flyers whose ground speeds decrease with increasing headwinds, and whose altitudes descend during flight [55,64]. To gain altitude, SLF begin flights from high points and ride air currents that provide lift [16,55]. In this study, peak height in trees corresponded with their peak movements during mating time (Figure 6D, Table 7), suggesting that mating and dispersal were both occurring during that time in the canopy. The positive relationship between height and step distance could also reflect walking and hopping between trees and vines in the contiguous canopy, but long-distance steps reflect a strategy by SLF to launch themselves from heights, allowing them to cover more distance in flight before descending.

Although this study described the frequency of SLF dispersing at different distances (Figure 4F), it was not clear whether mating typically occurs before or after dispersal. Two studies suggest that most dispersal occurs prior to mating, with some occurring after mating [55,56]. As gravid females are less flight-capable, there is only a brief opportunity after mating for females to disperse. The motivation to disperse may not necessarily be programmed at a specific time with respect to mating but could be triggered by depleting food resources [14,65], which is likely to occur around mating time when SLF are aggregating and feeding heavily. Supporting this hypothesis, the greatest dispersal events we observed were at the Trexler site in 2019 (see R_2_, Table 2), where the SLF density was 10-fold higher than in 2020, and their preferred hosts *A. altissima*, *J. nigra*, and *Vitis* were scarce in the vicinity of the release trees.

#### 4.3.3. Height as a Driver of the Perceived Sex Ratio Shift in Early-2

The movements of SLF depended on their sex and physiological status, and such differences in behavior between adult males and females over time may help explain some of the perplexing patterns of behavior observed in SLF. One such puzzling observation is that of their changing sex ratios [15,18,66,67]. At the beginning of Early-1, sex ratios on *A. altissima* tree trunks are typically close to 50% male, but Early-2 is marked by a sharp drop in sex ratio on their preferred host, as males suddenly become scarce. Where male SLF go during the observed shifts in sex ratio prior to mating has been a vexing question.

Several factors may contribute to observations of a sharply male-skewed sex ratio during Early-2. In this study, the most compelling evidence pointed to differing male and female movements to higher parts of trees. Remarkably in this study, even while the population at large maintained a 50% sex ratio, the eye level sex ratio over time strongly resembled that of SLF captured in previous studies in 1.3 m high traps on tree trunks (Figure 7C) [15,18,66,67,68]. Hence, the peculiar Early-2 disappearance of males documented in those studies may be an artifact of the sampling method which only captures the eye-level population. The sexual difference in Early-2 height may reflect males starting to position themselves in the canopy to intercept sexually receptive females arriving later. Observations of SLF mating have been limited [16]. Shifting preferences for tight aggregations and height over 8 m during Mid and Late-1 put the majority of mating in the canopy and help explain why descriptions of courtship and mating behavior have been so elusive.

#### 4.3.4. Distance Capacity, Dispersal, and Orientation Behavior

The most frequent step distance for tracked SLF was zero, confirming previous reports of SLF having sedentary tendencies [16,69]. However, the fraction of individuals that move have the capacity to transport themselves over 400 m (Figure 4F). As SLF females progressed through different stages, the frequency of their movement significantly decreased, but for males, there was an increase (Figure 3). The exception was during Mid when more than half of the steps of both sexes resulted in movement. The rate and distance of movements increased for females during Mid and Late-1, while for males they increased during Late-1 and Late-2 (Figure 4 and Figure 5). Although tracked SLF most commonly moved small distances, some moved very long distances starting around this time (Figure 2 and Figure 4).

Although attaching a tracking device to SLF may have affected the behavior and mobility of some, particularly the smaller males, it did not preclude some individuals from traveling unexpectedly long distances, longer than previously documented in the field [55,64]. Consequently, their average distance and rate of travel based on these experiments are likely underestimates of their true potential [55]. It does provide a baseline for what is possible.

It is not known whether tracked SLF traveled by walking or flying, but most step-distances observed in this study (Figure 2A,B) were shorter than the previous reports of individual flight distances of 10 to 50 m [16,70]. However, some outliers in this study traveled much longer than previously reported for individual SLF [16,70]. It has been observed that SLF carried by thermals to higher altitudes traveled longer distances [16,55]. Riding thermals may not be accidental, as this study found that SLF that climbed higher traveled longer distances during specific time periods. Climbing higher to improve the chances of making longer flights is likely a strategy used by SLF that maximizes their dispersal capabilities.

Dispersal and migration behavior in insects can be pre-programmed and triggered by specific abiotic factors such as time, photoperiod, temperature, and wind conditions [71], or can be triggered by biotic factors such as population crowding and food resource depletion [65]. Locusts famously migrate en masse, triggered by a combination of abiotic factors, such as decreasing light intensity, and biotic factors such as crowding [72,73,74], which, along with high population density, was found to be mediated by an aggregation pheromone [75]. SLF appear to share some of these features, including aggregation behavior, which may be guided by semiochemicals such as an aggregation pheromone or feeding-induced plant volatiles [15,61,62,63,76,77], and mass migration events at times when large SLF populations have depleted food resources [64,66]. Under this lens, it makes sense that in the low-density site used in 2020, only a small fraction of SLF traveled long distances, whereas in the high-density site used in 2019, several long-distance flights were recorded.

The movement observations in the current study reflect not individual flights or paths taken, but the distance between two points observed over a period of 1–3 days. This reflects a real-world dispersal distance over a defined period of time. As the time interval between observations was not always constant, due to both variations in when tracking was conducted and when individual SLF were located, the rate of SLF movement is perhaps a more meaningful parameter to describe SLF movement observed in this study.

Given that SLF prefer to take off and fly into the wind [64], it may be surprising that turning angles for RT-tagged adults were found to be random (Figure 2G). However, turning angle calculations certainly included walking and hopping steps, not only flight steps. Also, as wind directions were not measured either directly in the study area or at the time of flight, it is probable that the local wind direction influenced flight directions but correlations could not be detected given the experimental protocols used in the current study, as well as the numerous other factors that could be involved in the direction of their movement, such as the microclimate wind conditions and direction within the forest, relative locations of tall objects, host plants, aggregations, and forest edges. When analyzing the movement directions of RT-tagged SLF, non-random movement was detected (Figure 2H). Movement directions in 2019 showed non-random directions correlated with movement into the mean prevailing wind direction at the Trexler Nature Preserve. The northern release tree at Trexler was isolated on a hill in an open field and wind direction likely played a more prominent role in SLF movement directionality for this release point, particularly when compared to the other release trees in forested areas where the wind intensity was likely reduced. The observed movement into the wind there agrees well with the observations of Myrick and Baker [64] but the number of observations is low and the caveats listed above still apply.

Another variation in SLF movement parameters is seen in the observation that SLF flight capacity in tethered adults in the laboratory appears far greater than what we observed in the field [70]. This could be due to a number of factors such as the presence of suitable hosts, environmental conditions that limit flight distances, tag weight, or differences in motivation or exertion between tethered SLF that cannot land and free-flying SLF that must beat their wings in order to stay aloft. SLF also show a tendency to fly towards open areas when they are disturbed while feeding on *Ailanthus altissima*, but then quickly reorient and fly back towards tree lines [78]. This suggests that SLF have strong orientation mechanisms that bias movement towards their preferred hosts or prominent visual cues [70,79]. SLF orientation has a strong visual component and they have been observed to orient towards vertical poles [70,79] and skyscrapers [56]. They may be attracted to the light/dark contrast of the vertical objects providing a visual cue for the insects to navigate. In addition, SLF orientation is guided in part by olfactory cues such as host plant volatiles [77], honeydew excretions [76], and semiochemicals from other SLF [62]. Despite substantial evidence of sex-specific chemically-mediated orientation in SLF [76,77], and evidence to suggest SLF might use pheromones [62,63], no pheromones have been identified at this time. Recent evidence has been found that they also communicate using substrate vibrations (M.F.C. pers. obs.) [80]. Thus, their movements are guided by a complex array of sensory inputs at different spatial scales.

#### 4.3.5. Summarizing the Phenology of Tracked Movements for Adult SLF

Although our analysis of tracked SLF separately examines each parameter over time (movement, height, aggregation, sex ratio, courtship), a combination of these parameters forms each data point, thus the parameters should be considered holistically (Table 7).

In Early-1, females made more frequent, but short-distance movements (averaging less than 5 m), as they located suitable hosts and sites for feeding. During this time, female step distances were strongly correlated with their height in trees, and SLF were not in tight aggregations yet. Of those that moved, the overall distance and rate of movement were low, so Early-1 movements were short. They were equally likely to be above and below 8 m, and females were just as likely to be found at eye level as males. Concurrently, the frequency of male movement was at its lowest of the season.

During Early-2, males, but not females, started shifting their vertical positions to above eye level, which may contribute to the perceived sex ratio shift frequently observed up to 2 m high, where males are perceived to become scarce. Only about one-third of female steps and one-quarter of male steps resulted in movement to new positions, and again, these movements were relatively short distances of a few meters.

During Mid, both sexes became more active as movement from one position to another, regardless of distance, occurred more often than not for both sexes. This coincided with increased aggregation, residing mostly above 8 m, the onset of mating, and increased distance (males) or rate (females) moved.

Late-1 bore an intensification of nearly all parameters except for frequency of movement. While only males remained predominantly above 8 m, distance, rate, the correlation between height and distance, the shift above eye level, and aggregation behavior all intensified for both sexes. Although females moved most frequently during Early-1 and Mid, those that moved during Mid and particularly Late-1 went the longest distances and fastest rates. Conversely, while males moved most frequently during Mid, those that moved went increasingly longer distances and faster rates as the season progressed to Late-2. Both sexes started orienting to trees with tight aggregations during Mid, presumably for mate location and courtship. Orientation to trees with these aggregations peaked during Late-1, lasting 2.5 weeks for males, and 2 weeks for females which started half a week after males. Thus, peak mating seemed to extend into the first few days of Late-2. Similarly, although only a small number of tagged SLF were observed engaging in courtship behavior, most of these observations occurred during Late-1. Therefore, although mate-location and mating began during Mid, courtship, and mating peaked during Late-1.

During Late-2, we observed a sharp reduction in movement by females while male movements increased (Figure 3B, Table 7). The height of both sexes again was centered around 8 m and aggregating diminished. After mating, females continue feeding as their ovaries develop and their abdomens swell, hindering flight [55], and they may still be courted by virgin males even as some descend to lower heights (Figure 7A). A fraction of mated females may mate again, but male genitalia remain in the female reproductive tract, limiting males to only one mate and resulting in post-copulation mortality [81]. Oviposition begins roughly 1 week after mating, and females may deposit multiple egg masses [82].

## 5. Conclusions

This study demonstrates the feasibility of tracking SLF with both RT and HR. Adult movements were best followed using RT while the lower mass and size of HR was best suited to tracking nymphs. Tracking adult SLF over the course of two field seasons revealed timing and insights into SLF movement parameters such as frequency of movement, step distance, rate, height, host preference, and directionality. In particular, 90% of SLF reside above eye level, and the average height was 8 m. Observation of SLF height in trees appears to explain that cryptic “missing males” are located higher in trees and are therefore hidden from view. And finally, plant preferences varied by sex.

## Figures and Tables

**Table 1:** Tracking parameters, over two years, for spotted lanternfly (SLF) *L. delicatula* adults affixed with either an RT or HR tag and tracked until the last time a tag was located, either detached, on a dead SLF, or on a live SLF that was subsequently never located again (lost). The total distance traveled and rate of travel for SLF that moved are also provided for females and males separately. Recovery frequencies were compared using the chi-square test and movement parameters were compared using *t*-test, or Wilcoxon test if not normally distributed. Means are followed by ±standard error, and values in the same row followed by different letters differed significantly (*p* < 0.05).

Measurement	Radio Telemetry	Harmonic Radar
No. recovered/total N tagged SLF (%)	148/185 (80%)	33/60 (55%)
No. not recovered (% of total) ^a^	37 (20%) ^a^	27 (45%) ^b^
No. recovered > 0 m (% of recovered) ^a^	116 (78%) ^a^	20 (61%) ^a^
No. recovered < 3 m (% of recovered) ^a^	75 (51%) ^a^	19 (58%) ^a^
No. recovered 3+ m (% of recovered) ^a^	73 (49%) ^a^	14 (42%) ^a^
Total no. steps	535	62
No. steps with movement (% of tot.)	226 (42%)	23 (37%)
No. steps with duration = 1–3 d (% of tot.)	471 (88%)	42 (68%)
No. 1–3 d steps with movement (%)	183 (81%)	6 (26%)
Mean (max) time tracked (d) ^b^	7.3 ± 0.7 (37) ^a^	5.9 ± 1 (22) ^a^
Mean (max) no. observations per SLF ^b^	3.7 ± 0.4 (21) ^a^	2.2 ± 0.4 (11) ^b^
Mean (max) total dist. per SLF (m) ^b,c^	20.6 ± 5.4 (434) ^a^	12.5 ± 3.7 (60) ^a^
Mean (max) rate of movement (m/d) ^b^	1.9 ± 0.4 (144.7) ^a^	0.7 ± 0.2 (5.2) ^b^
N tagged SLF females (N moved)	91 (65)	34 (12)
Mean (max) total female distance (m) ^b^	31.8 ± 9 (434) ^a^	13.4 ± 6 (60) ^a^
Mean (max) female rate (m/d) ^b^	5.9 ± 1.3 (145) ^a^	1.8 ± 0.5 (5) ^b^
N tagged SLF males (N moved)	94 (51)	26 (8)
Mean (max) total male distance (m) ^b^	6.3 ± 1.3 (57) ^a^	11.2 ± 3.9 (28) ^a^
Mean (max) male rate (m/d) ^b^	2.2 ± 0.3 (11) ^a^	2.8 ± 0.6 (5) ^a^

^a^ Chi-square test; ^b^ *t*-test; ^c^ SLF that left their release tree.

**Table 2:** The frequencies (and number of steps) that all tracked SLF females (F) and males (M) were located in different habitat types. Movements by release habitat in three habitat types (field, forest edge, and inner forest) are shown. Those that were found in the same habitat type as where they were released are shown in bold, for each of the five release sites (R_1_–R_5_), and for both years and technologies.

Release Location—Habitat		Frequency Found (n)			Total Steps
	Sex	Field	Forest Edge	Inner Forest	
R_1_—Tree island in field	F	**100% (33)**	0%	0%	33
	M	**100% (32)**	0%	0%	32
R_2_—Forest edge	F	9.4% (3)	37.5% (12)	**53.1% (17)**	32
	M	16.1% (5)	**58.1% (18)**	25.8% (8)	31
R_3_—Inner forest	F	9.4% (12)	18.8% (24)	**71.9% (92)**	128
	M	0%	12.2% (14)	**87.8% (101)**	115
R_4_—Inner forest	F	0%	7.9% (11)	**92.1% (129)**	140
	M	0%	0%	**100% (65)**	65
R_5_—Forest edge	F	0%	**100% (26)**	0%	26
	M	0%	**100% (5)**	0%	5

**Table 3:** Movement parameters for SLF adults tracked with HR over two years using tags of different materials. In 2019, each adult was affixed with a platinum antenna and released at Trexler Nature Preserve. In 2020, each adult was affixed with a nitinol antenna and released at Beaver Brook Nature Preserve. Values followed by different letters are significantly different by *t*-test (*p* < 0.05).

Measurement	Platinum	Nitinol
Mean total distance (m)	5.8 ± 4 a	9.7 ± 4 a
Max. total distance (m)	60.0	50.2
Mean time tracked (d)	4.0 ± 1 a	8.2 ± 2 a
Max time tracked (d)	18	22
Mean obs./SLF	1.2 ± 0.1 a	2.7 ± 0.7 b
Max. obs./SLF	2	11
Mean rate (m/d)	2.5 ± 0.8 a	1.9 ± 0.4 a
Max. rate (m/d)	5.0	5.2
N SLF	43	17

**Table 4:** Movement parameters for SLF nymphs tracked with HR in a corn field. Values followed by different letters are significantly different by *t*-test (*p* < 0.05).

Measurement	Nitinol	Tungsten
Mean total distance (m)	16 ± 4 a	8 ± 3 a
Max. total distance (m)	27.6	17.7
Mean time tracked (d)	5.07 ± 0.01 a	3 ± 1 b
Max. time tracked (d)	5.09	5.10
Mean obs./SLF	3 ± 0 a	2 ± 1 a
Max obs./SLF	3	3
Mean rate (m/d)	4.3 ± 0.6 a	6 ± 2 a
Max. rate (m/d)	9.9	19.0
N	5	5

**Table 5:** Movement parameters for SLF nymphs tracked with HR at the corn field/forest interface. Values followed by different letters are significantly different by *t*-test (*p* < 0.05).

Measurement	Nitinol	Tungsten
Mean total distance (m)	3.0 ± 0.3 a	0.8 ± 0.2 b
Max. total distance (m)	4	1.3
Mean time tracked (d)	1.5 ± 0.2 a	0.8 ± 0.3 a
Max. time tracked (d)	1.83	1.83
Mean obs./SLF	3.4 ± 0.4 a	2.0 ± 0.5 a
Max obs./SLF	4	4
Mean rate (m/d)	2.2 ± 0.6 a	1.8 ± 0.9 a
Max. rate (m/d)	4.7	5.5
N	5	5

**Table 6:** Movement parameters for nymphal SLF tracked with HR compared between micro-locations (in corn field “Corn” and at the corn field/forest interface “Edge”). Tag types have been combined for this analysis. Values followed by different letters are significantly different by *t*-test (*p* < 0.05).

Measurement	Corn	Edge
Mean total distance (m)	12 ± 3 a	1.9 ± 0.4 b
Max. total distance (m)	27.6	4.0
Mean time tracked (d)	3.9 ± 0.6 a	1.2 ± 0.2 b
Max. time tracked (d)	5.1	1.83
Mean obs./SLF	2.5 ± 0.3 a	2.7 ± 0.4 a
Max obs./SLF	3	4
Mean rate (m/d)	3.3 ± 0.4 a	2.1 ± 0.5 b
Max. rate (m/d)	5.4	5.5
N	10	10

**Table 7:** A summary showing the timing of activities of tagged SLF adult females (F) and males (M). A single X indicates the stage with the activity. If more than one stage had the activity, XX represents the stage with more intense activity.

		Timing of Peak Intensity *				
Activity of Tagged SLF	Sex	Early-1	Early-2	Mid	Late-1	Late-2
Frequent movement (>50%)	F	XX		X		
(Figure 3B)	M			XX		X
Greatest distances per SLF	F				X	
(Figure 4A)	M			X		
Longest step distances	F				X	
(Figure 4D,E)	M				X	XX
Fastest rates of movement	F			X	XX	
(Figure 5B,C)	M				X	XX
Distance increases with height	F	X			X	
(Figure 6D)	M			X	X	
Shift above eye level (>85%)	F				X	
(Figure 7)	M		X		X	
Mostly above 8 m high (>50%)	F			X		
(Figure 6F)	M			XX	X	
Found near tight aggregations	F			X	XX	X
(Figure 9)	M			X	XX	X
Courtship observed	Both			X	XX	X

* The start of each stage is defined by the observed event: Early-1, adults emerge; Early-2, observed sex ratio shifts making males seem scarce; Mid, first observation of mating in the field; Late-1, first observation of oviposition in the field; Late-2, two weeks following first oviposition.

## Data Availability

The raw data supporting the conclusions of this article will be made available by the authors, without undue reservation.
